# Cholesterol 25‐Hydroxylase Enhances Myeloid‐Derived Suppressor Cell (MDSC) Immunosuppression via the Stimulator of Interferon Genes (STING)‐Tank‐Binding Kinase 1 (TBK1)‐Receptor‐Interacting Protein Kinase 3 (RIPK3) Pathway in Colorectal Cancer

**DOI:** 10.1002/mco2.70411

**Published:** 2025-09-27

**Authors:** Dongqin Zhou, Yu Chen, Xudong Liu, Juan He, Luyao Shen, Yongpeng He, Jiangang Zhang, Yu Zhou, Nan Zhang, Yanquan Xu, Juan Lei, Ran Ren, Huakan Zhao, Xianghua Zeng, Yongsheng Li

**Affiliations:** ^1^ The Second Affiliated Hospital and Yuying Children's Hospital of Wenzhou Medical University Wenzhou Zhejiang China; ^2^ Department of Medical Oncology Chongqing University Cancer Hospital Chongqing China; ^3^ School of Medicine Chongqing University Cancer Hospital, Chongqing University Chongqing China; ^4^ Department of Clinical Laboratory Air Force Medical Center Beijing China; ^5^ Chongqing Key Laboratory of Translational Research for Cancer Metastasis and Individualized Treatment Chongqing University Cancer Hospital & Chongqing Cancer Institute & Chongqing Cancer Hospital Chongqing China; ^6^ Clinical Medicine Research Center Xinqiao Hospital, Army Medical University Chongqing China

**Keywords:** cholesterol metabolism, cholesterol 25‐hydroxylase, colorectal cancer, myeloid‐derived suppressor cells, stimulator of interferon genes, tumor immunosuppression, 25‐hydroxycholesterol

## Abstract

Myeloid‐derived suppressor cells (MDSCs) represent a significant immunosuppressive population within the tumor microenvironment of colorectal cancer (CRC). Their activity has been strongly associated with the reprogramming of cholesterol metabolism, although the underlying mechanisms remain unclear. To investigate this, we generated myeloid‐specific cholesterol 25‐hydroxylase (CH25H) knockdown mice and differentiated bone marrow cells from wild‐type (WT) or *Ch25h* ^f/f^ Lyz2^Cre^ mice into MDSCs, subsequently treating them with 25‐hydroxycholesterol (25HC). Immune function was evaluated using flow cytometry, Western blotting, and real‐time polymerase chain reaction (PCR). Our findings indicated that CH25H and its metabolite 25HC were significantly upregulated in CRC‐associated MDSCs. The loss of CH25H impaired their immunosuppressive capacity by reducing arginase‐1 (ARG1) expression, an effect that was restored by 25HC supplementation. Mechanistically, 25HC suppressed the activation of the cyclic guanosine monophosphate–adenosine monophosphate synthase–stimulator of interferon genes (cGAS–STING) pathway and the downstream tank‐binding kinase 1 (TBK1). TBK1 formed a complex with receptor‐interacting protein kinase 3 (RIPK3), thereby repressing ARG1 expression through phosphorylation‐dependent signaling. Collectively, these findings reveal a previously unrecognized CH25H–25HC–STING axis in MDSC‐mediated immune regulation and suggest that targeting cholesterol metabolism may provide a promising therapeutic strategy for CRC immunotherapy.

## Graphical abstract

A schematic diagram outlines the mechanism by which CH25H enhances the immunosuppressive function of CRC‐infiltrating MDSCs. CH25H in MDSCs inhibits the phosphorylation activation of STING and its downstream effectors, TBK1 and RIPK3, within the endoplasmic reticulum, mediated by its metabolite, 25HC. This inhibition leads to increased expression of ARG1 in MDSCs, thereby enhancing their immunosuppressive activity and facilitating immune evasion in CRC tumor microenviroment. Conversely, a specific deficiency of CH25H disrupts this cascade of changes, resulting in decreased ARG1 expression and diminished immune function of MDSCs.

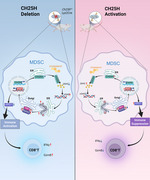

## 1 Introduction

Colorectal cancer (CRC) is among the most prevalent malignant tumors of the human digestive system. Immunotherapy targeting programmed cell death‐1 (PD‐1) and its ligand programmed cell death ligand‐1 (PD‐L1) has significantly improved survival rates in various cancer types. However, PD‐1 monotherapy exhibits limited efficacy in the majority of CRC patients with microsatellite stability (MSS) or proficient mismatch repair (pMMR), and is therefore not recommended for metastatic CRC [1]. Consequently, understanding the mechanisms underlying the limited response of CRC to immunotherapy has become a critical research priority. Additionally, exploring strategies to enhance the therapeutic effectiveness of immunotherapy in CRC is of great clinical significance.

Immunosuppression plays a critical role in the initiation and progression of tumors, enhancing tolerance to immunotherapy directed against these malignancies [2]. The immunosuppression observed during cancer development and progression results from the coordinated efforts of various cell types, particularly myeloid‐derived suppressor cells (MDSCs) [3]. MDSCs are the most abundant precursor cells in the bone marrow (BM), migrating to peripheral tissues and the tumor microenvironment (TME), where they express elevated levels of arginase 1 (ARG1), inducible nitric oxide synthase (iNOS/NOS2), PD‐L1, and reactive oxygen species (ROS), among other immunosuppressive molecules [4, 5]. They inhibit T cell proliferation and antitumor functions while promoting tumor metastasis and immune resistance [4, 6, 7], and are also known to trigger epithelial‐to‐mesenchymal transition (EMT) [8], thereby accelerating CRC progression. Our previous studies have revealed that MDSCs within the TME of CRC are central mediators of immune evasion [9, 10, 11]. Consequently, targeting MDSCs may represent a promising strategy to enhance the antitumor immune response in CRC.

In recent years, key regulators of MDSCs activation have been identified, including proinflammatory factors such as interferon‐gamma (IFN‐γ), interleukin (IL)‐1β, IL‐4, IL‐13, and prostaglandin E_2_ (PGE_2_), as well as signaling pathways involving signal transducer and activator of transcription 6 (STAT6), nuclear factor kappa‐B (NF‐κB), STAT1, and endoplasmic reticulum (ER) stress, along with lipid metabolism pathways [12]. Our previous studies have demonstrated a close link between the reprogramming of cholesterol metabolism and the differentiation of hematopoietic progenitor cells (HPCs) into MDSCs, as well as the regulation of immune functions in these cells [10, 11]. Cholesterol metabolism encompasses several steps, including the biosynthesis of cholesterol from its precursors, the uptake and efflux of cholesterol, its esterification, and oxidation [13, 14]. Cholesterol 25‐hydroxylase (CH25H) serves as a critical enzyme that catalyzes the oxidation of cholesterol to 25‐hydroxycholesterol (25HC). This metabolic pathway is crucial for various cellular processes, including antiviral defense, inflammatory responses, signal transduction, and tumor metastasis [15]. Notably, it has been reported that 25HC activates the STAT6‐dependent signaling pathway via adenosine monophosphate (AMP)‐activated protein kinase α (AMPKα), thereby promoting the expression of ARG1 in tumor‐associated macrophages (TAMs) [16]. However, the role of the CH25H metabolic pathway in the functional regulation of tumor‐associated MDSCs remains largely unexplored.

Our previous research indicates a positive correlation between CH25H and 25HC regarding the immunosuppressive properties of MDSCs [10]. This correlation suggests that the metabolic reprogramming of 25HC may affect the regulatory functions of MDSCs. In this study, we aim to investigate the role and underlying mechanisms by which CH25H regulates the function of MDSCs and the progression of CRC, thereby providing new insights for the immunotherapy of CRC.

## 2 Results

### 2.1 CH25H Is Upregulated in CRC‐Associated MDSCs

**FIGURE 1:**
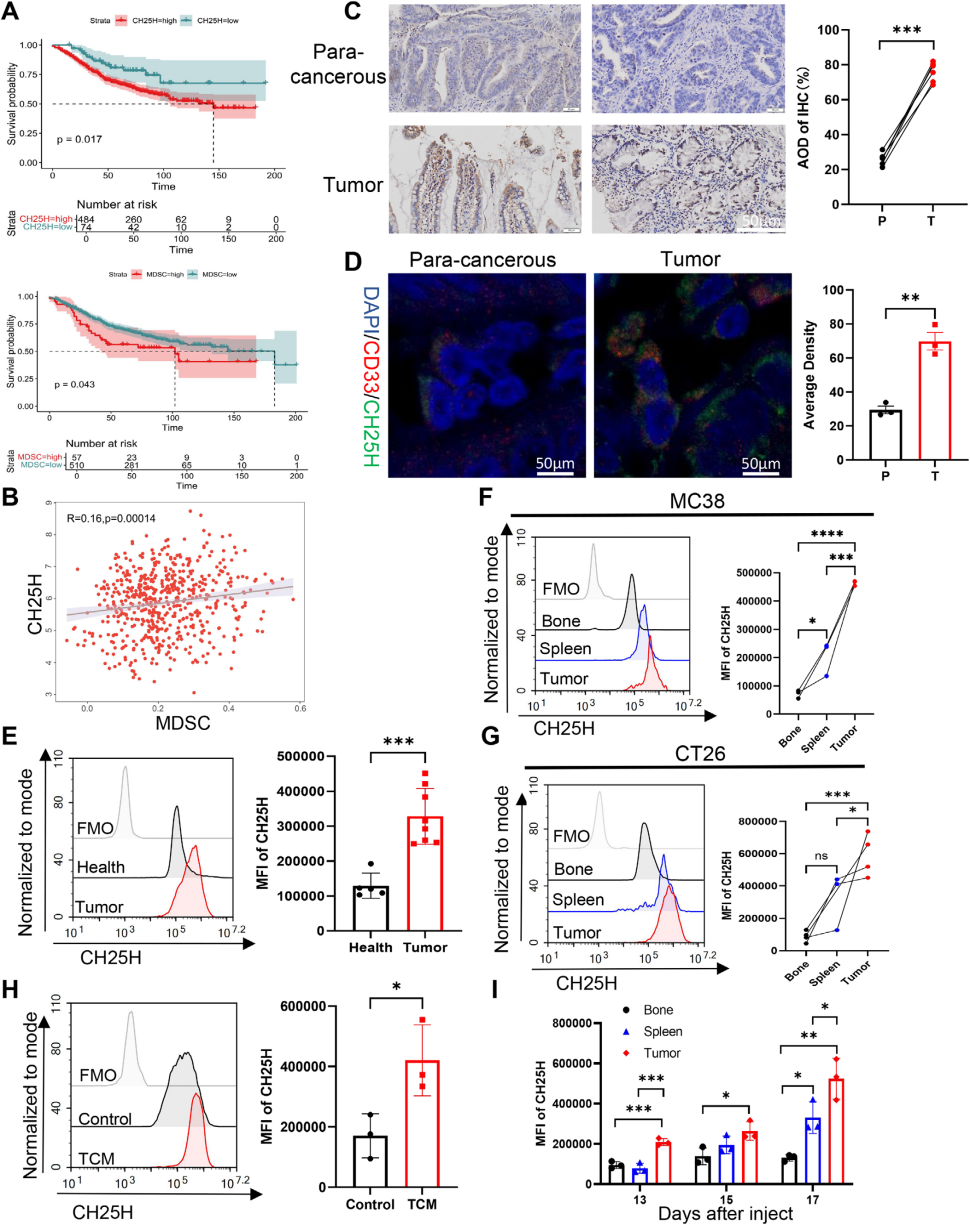
The abundance of CH25H is increased in tumor‐infiltrated MDSCs. (A, B) The CH25H and MDSC risk‐prognostic models were assessed using four independent datasets sourced from the GEO database: GSE39582, GSE17538, GSE87211, and GSE94104. The analysis of CH25H and MDSC scores was conducted through Spearman's correlation analysis, with results visualized using scatter plots. (C) IHC tests were conducted to assess differences in CH25H expression levels and the AOD of the percentage of positive area in paracancerous (P) and tumor (T) tissues of CRC patients (*n* = 8). (D) IF was employed to measure discrepancies in CH25H expression levels in MDSCs from cancerous and paraneoplastic tissues of CRC patients (*n* = 3). (E) The levels of CH25H in peripheral blood MDSCs of CRC patients and healthy donors (*n* = 5 or 8) were analyzed. FMO: fluorescence minus one; MFI: mean fluorescence intensity. (F) The CH25H levels in bone marrow, spleen, and tumor tissue MDSCs were examined in the C57BL/6 male mouse subcutaneous (*s.c*.) MC38 tumor model (*n* = 3), using bone marrow MDSCs as the control group. (G) The CH25H levels in bone marrow, spleen, and tumor tissue MDSCs were also evaluated in the *s.c*. CT26 model using C57BL/6 male mice (*n* = 3), using bone marrow MDSCs as the control group. (H) The levels of CH25H after in vitro treatment of MDSCs with MC38‐TCM (RPMI1640 medium: TCM = 1:1) were assessed (*n* = 3), the RPMI1640 medium treatment group was set as the control group. (I) Continuous monitoring of CH25H levels in MDSCs across different tissues (bone marrow, spleen, and tumor) in the *s.c*. CT26 tumor model was performed at various time points postloading (*n* = 3), using bone marrow MDSCs as the control group. Data are expressed as mean ± SEM values. Comparisons to controls are indicated, with ns denoting no significant difference, *p < 0.05, **p < 0.01, ***p < 0.001.

We analyzed multiple cohorts of CRC patients data from the gene expression omnibus (GEO) database, employing risk‐prognostic modeling and immune infiltration analysis based on immune cell biomarkers. Our findings indicated that both high expression levels of CH25H and a high percentage of MDSCs infiltration were significantly associated with poor prognosis (Figure 1A). Furthermore, correlation analysis revealed a positive association between the CH25H score and the MDSCs characterization score (Figure 1B), corroborating our previous study. Additionally, another report demonstrated that the messenger ribonucleic acid (mRNA) levels of CH25H were higher in tumor‐infiltrating MDSCs compared to spleen‐infiltrating MDSCs [16]. This prompted us to investigate whether CH25H levels were elevated in MDSCs of CRC patients. Initially, IHC staining of paraffin sections from surgical pathology specimens of eight CRC patients revealed that the average optical density (AOD) percentage of the positive area was significantly higher in histopathological sections of tumor (T) compared to paracancerous (P) adjacent normal tissue. This indicated a markedly greater accumulation of CH25H in cancerous tissues (Figure 1C), aligning with our findings of a distinct increase of CH25H in CRC tumor tissues as analyzed using the GEO database (Figure S1A). Subsequently, we performed immunofluorescence (IF) staining on these paraffin sections following dewaxing treatment. Through a literature review, we identified cluster of differentiation 33 (CD33) as the signature molecule for localizing MDSCs [17, 18, 19]. The final results demonstrated that the number of MDSCs was significantly increased in tumor tissues compared to paraneoplastic tissues, with a concurrent upregulation of CH25H expression in MDSCs (Figure 1D). Moreover, CH25H levels were markedly elevated in peripheral blood MDSCs (CD11b^+^ human leukocyte antigen‐DR [HLA‐DR]^−^) from CRC patients compared to healthy donors (Figure 1E).

Subsequently, we conducted further testing and validation using murine models. We utilized two CRC cell lines, MC38 and CT26, to establish subcutaneous tumor models in C57BL/6 wild‐type (WT) male mice and Balb/C WT male mice, respectively. The mice were euthanized when the tumor volume reached 1000–1500 mm^3^, and flow cytometry was performed to assess CH25H levels in MDSCs characterized as CD11b^+^Gr1^+^. Notably, we observed a significantly elevated expression of CH25H in tumor‐infiltrating MDSCs compared to those derived from BM or spleen sources (Figure 1F,G). Additionally, we employed three other tumor cell lines—B16‐F10 (Supporting Information Figure 1B), Hepa1‐6 (Supporting Information Figure 1C), and LLC (Figure S1D)—to establish subcutaneous mouse tumor models. All models exhibited consistent findings, demonstrating higher levels of CH25H expression in tumor‐associated MDSCs. Furthermore, CH25H expression was also found to be upregulated in the MDSCs treated with MC38 tumor‐conditioned medium (TCM; Figure 1H). Moreover, our sequential assays in Balb/C WT male mice, utilizing a subcutaneous tumor model, revealed a progressive increase in CH25H expression in MDSCs throughout the tumor growth phase of CRC, consistently surpassing levels observed in BM and spleen MDSCs (Figure 1I). Collectively, these findings indicate that the TME significantly induces elevated CH25H expression in MDSCs, which may enhance their immunosuppressive capabilities.

### 2.2 CH25H‐Derived 25HC Enhances MDSC‐Mediated Immunosuppression

**FIGURE 2:**
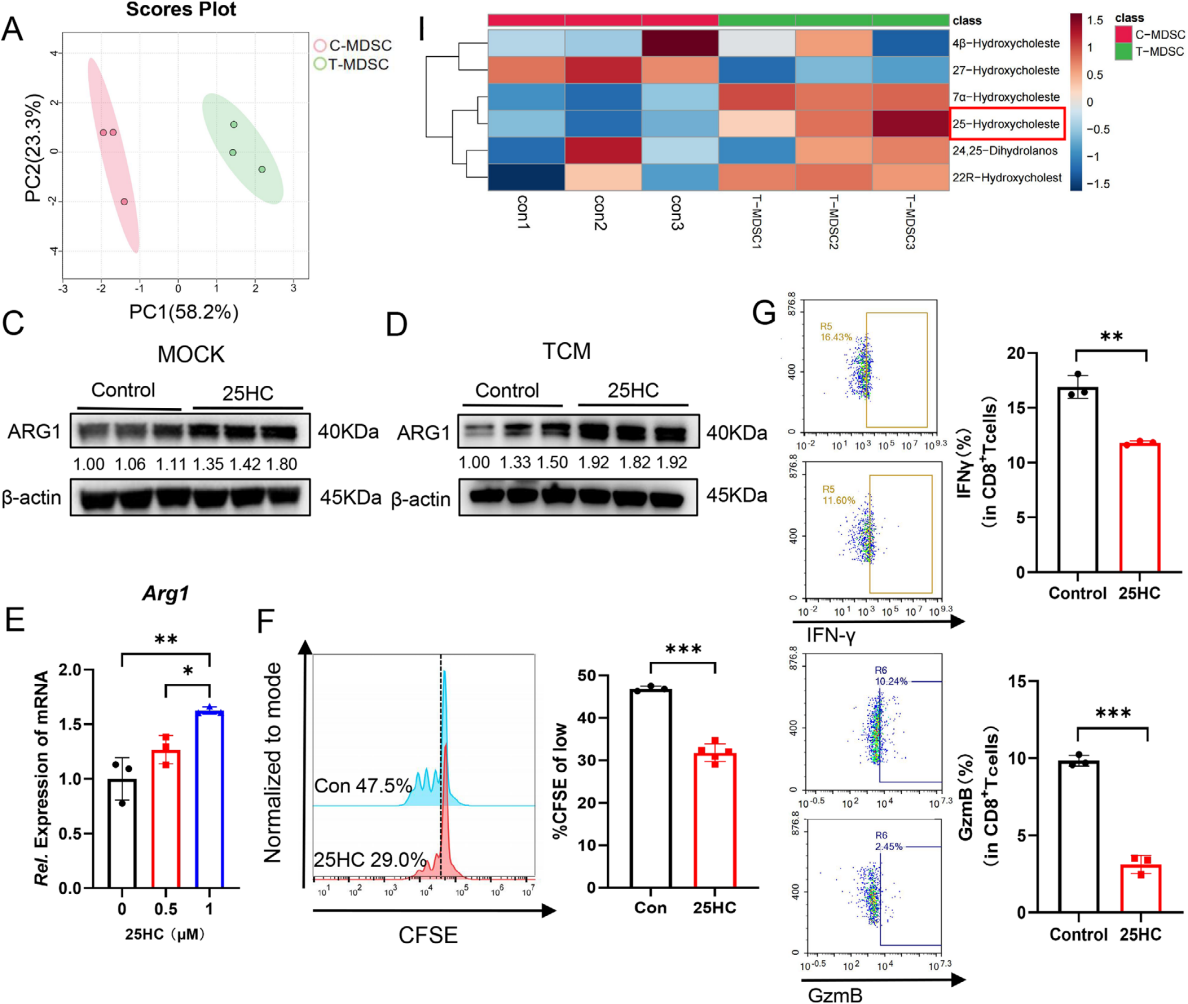
The immunosuppressive function of tumor‐associated MDSCs is regulated by CH25H metabolism of cholesterol, leading to the generation of 25HC. (A) Principal component analysis (PCA) of BM‐MDSCs in subcutaneously (s.c.) MC38‐loaded and tumor‐free mice (*n* = 3). PC1: Principal component 1. (B) UPLC–MS/MS was employed to detect the relative amounts of free cholesterol‐related metabolites in BM‐MDSCs from the samples in panel (A), using tumor‐free mice MDSCs as the control group. (C) The protein level of ARG1 was measured following in vitro induction of MDSCs treated with 25HC (0.5 µM) for 6 h, using DMSO as a control (*n* = 3). (D) The protein level of ARG1 was assessed after treatment with 25HC (0.5 µM, 6 h) in MDSCs induced in vitro and subsequently combined with MC38‐TCM treatment, using DMSO as the control (*n* = 3). (E) The relative mRNA levels of ARG1 in MDSCs were analyzed after treatment with various concentrations of 25HC (0, 0.25 µM, 0.5 µM) for 6 h, using DMSO as the control (*n* = 3). (F) T cell proliferation was evaluated after treatment with 25HC (0.5 µM, 6 h) combined with MC38‐TCM in MDSCs cocultured with carboxyfluorescein succinimidyl ester (CFSE)‐labeled CD8^+^ T cells at a ratio of 1:3 for 72 h (*n* = 3 or 5), using DMSO as the control. (G) MDSCs treated with 25HC (0.5 µM, 6 h), using DMSO as the control, combined with MC38‐TCM were cocultured with CD8^+^ T cells (ratio 1:3) that were stimulated with a Cell Activation Cocktail (including Brefeldin A) and Golgistop for 48 h, followed by a T cell activation function assay (*n* = 3). Data are expressed as mean ± SEM values. Statistical significance is indicated, with ns representing no difference, *p < 0.05, **p < 0.01, ***p < 0.001.

To investigate the role of CH25H upregulation in tumor‐associated MDSCs during the disease procession, we assessed the levels of 25HC in BM‐MDSCs from mice subcutaneously implanted with MC38 tumors compared to unloaded controls using ultra performance liquid chromatography mass spectrometry (UPLC–MS/MS; Figure 2A). A significant increase in 25HC levels was observed (Figure 2B), and similar results were obtained from uncharged BM‐MDSCs treated with MC38TCM in vitro (Figure S2A). These findings indicate that CH25H may influence MDSCs function through its cholesterol oxidation product, 25HC. Furthermore, we noted an enhanced expression of ARG1, a key immunosuppressive molecule associated with MDSCs, following 25HC treatment, while the levels of iNOS, PD‐L1, and ROS did not change significantly (Figures 2C, S2B–D). Additionally, the protein expression of ARG1 was also found to be upregulated in MDSCs treated with 25HC in the presence of MC38‐TCM (Figure 2D). Moreover, the mRNA transcript levels of *Arg1* increased in a concentration‐dependent manner in response to 25HC treatment (Figure 2E), whereas no significant differences were observed for other immunosuppressive molecules, including PD‐L1 and iNOS (Figure S2E).

TAMs exert their effects by facilitating cholesterol efflux and enhancing macrophage immunosuppression. The deletion of genes encoding ATP‐binding cassette (ABC) transporter proteins, which mediate cholesterol efflux (such as ABCA1 and ABCG1), has been shown to reverse the tumor‐promoting functions of TAMs and slow tumor progression [20]. Quantitative polymerase chain reaction (qPCR) results indicated that increasing concentrations of 25HC corresponded with a gradual increase in the expression of *Abca1* (Figure S2E), providing further evidence that 25HC effectively enters cells and exerts its functions. We concluded that 25HC enhances the immunosuppressive properties of MDSCs by upregulating the expression of ARG1. Given the robust inhibitory effects of MDSCs on CD8^+^ T cells [6, 7, 21], we isolated 25HC‐treated MDSCs induced by the MC38‐TCM and cocultured them with CD8^+^ T cells stimulated with anti‐CD3 and anti‐CD28 antibodies. As expected, the proliferation of CD8^+^ T cells was significantly suppressed by 25HC treatment compared to the control group, as evidenced by reduced expression levels of IFNγ, granzyme B (GzmB), and tumor necrosis factor‐α (TNFα; Figures 2F,G and S2F). Collectively, these findings suggest that CH25H may influence MDSCs through its metabolically produced 25HC, thereby enhancing their immunosuppressive function by promoting ARG1 expression.

### 2.3 Reversal of MDSC Immunosuppression by 25HC in CH25H‐Deficient Mice

**FIGURE 3:**
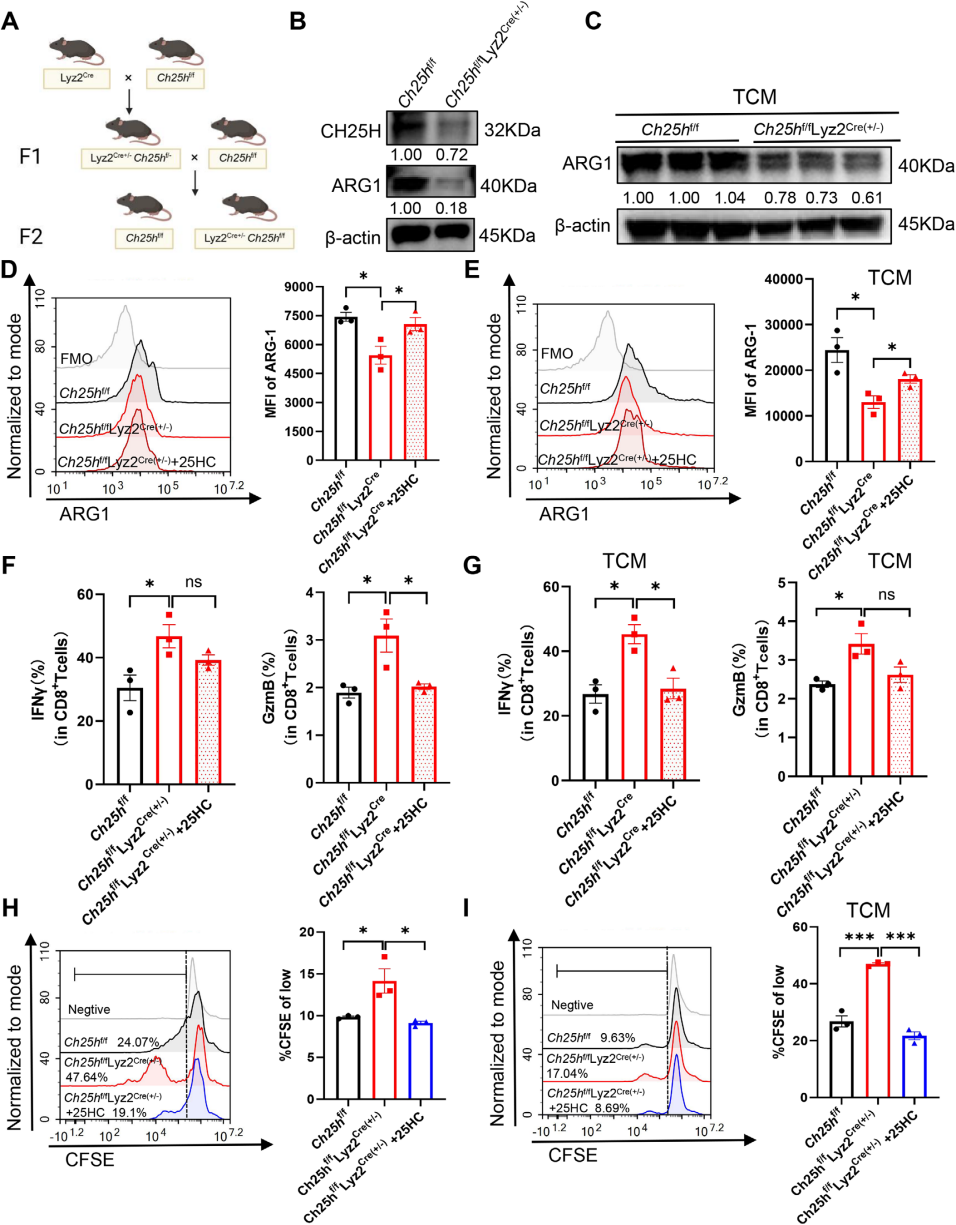
The myeloid deletion of CH25H reduces the immunosuppressive effects of MDSCs, an effect that can be reversed by the addition of 25HC. (A) A graphical representation illustrating the establishment of a myeloid CH25H‐deficient mouse model. (B) The protein level of ARG1 was measured after in vitro treatment of *Ch25h* ^f/f^Lyz2^Cre(±)^ MDSCs (using *Ch25h^f/f^* MDSCs as the control) in RPMI1640 medium (*n* = 3). (C) The protein level of ARG1 following MC38‐TCM treatment of *Ch25h^f/f^* Lyz2^Cre(±)^ MDSCs (using *Ch25h^f/f^* MDSCs as the control) was assessed (*n* = 3). (D) The protein level of ARG1 was evaluated after the induction of *Ch25h^f/f^* Lyz2^Cre(±)^ MDSCs (using *Ch25h^f/f^* MDSCs as the control) in vitro, followed by backfilling with 25HC (0.5 µM, 6 h) (using the same volume of DMSO as the control condition; *n* = 3). (E) The protein level of ARG1 was analyzed after MC38‐TCM treatment of *Ch25h^f/f^* Lyz2^Cre(±)^ MDSCs (using *Ch25h^f/f^* MDSCs as the control), with the addition of 25HC (using the same volume of DMSO as the control condition; *n* = 3). (F) The functional activation level of CD8^+^ T cells was detected after the induction of *Ch25h^f/f^* Lyz2^Cre(±)^ MDSCs (using *Ch25h^f/f^* MDSCs as the control) in vitro and subsequent rebinding of 25HC in coculture with activation‐stimulated CD8^+^ T cells for 48 h (*n* = 3). (G) MC38‐TCM treatment in vitro induced *Ch25h^f/f^* Lyz2^Cre(±)^ MDSCs (using *Ch25h^f/f^* MDSCs as the control), which were then backfilled with 25HC and cocultured with activation‐stimulated CD8^+^ T cells for 48 h to examine the level of CD8^+^ T cell activation (*n* = 3). (H) T cell proliferation was assessed following the induction of *Ch25h* ^f/f^Lyz2^Cre(±)^ MDSCs (using *Ch25h^f/f^* MDSCs as the control) in vitro. These cells were subsequently backfilled with 25HC and cocultured with CD8^+^ T cells for 72 h (*n* = 3). (I) T cell proliferation was evaluated after treatment with MC38‐TCM, which induced *Ch25h* ^f/f^Lyz2^Cre(±)^ MDSCs that were also backfilled with 25HC and cocultured with CD8^+^ T cells for 72 h (*n* = 3). Data are expressed as mean ± SEM values. Comparisons with controls are indicated, where ns denotes no significant difference, *p < 0.05, **p < 0.01, ***p < 0.001.

To comprehensively investigate the role of CH25H in CRC‐associated MDSCs, we successfully generated conditioned *Ch25h* knockdown C57BL/6 mice using the Lyz2^Cre/LoxP^ recombination system (Figure 3A). UPLC–MS/MS analysis of BM‐MDSCs from mice with negative genotypic identification (Figure S3A) demonstrated an increased cholesterol content and a decreased level of the metabolite 25HC compared to *Ch25h* ^f/f^ control mice (Figure S3B). The efficiency of the knockdown was further confirmed through Western blot analysis (Figures 3B and S3C). Compared to *Ch25h* ^f/f^ mice, both Western blot and flow cytometry analyses revealed a significant reduction in ARG1 expression in MDSCs following *Ch25h* myeloid‐specific knockdown (Figure 3B,D). Conversely, the addition of 25HC reversed this suppression, resulting in increased ARG1 expression (Figure 3D). These findings were replicated following treatment with MC38‐TCM (Figure 3C,E). Additionally, coculture of *Ch25h* ^f/f^Lyz2^Cre(±)^ MDSCs resulted in an upregulation of IFNγ and GzmB expression, as well as an enhancement in the proliferative capacity of CD8^+^ T cells compared to *Ch25h* ^f/f^ mice, which mitigated the inhibitory effects on CD8^+^ T cells. However, this effect was further inhibited and downregulated by the addition of 25HC (Figure 3F,H), with similar results observed after treatment with MC38‐TCM (Figure 3G,I). In conclusion, our results indicate that ARG1 expression is downregulated and the immunosuppressive activity of MDSCs is inhibited as a result of *Ch25h* myeloid‐specific knockdown. However, these effects can be restored by the addition of 25HC in a complementary manner.

### 2.4 Inhibition of the cGAS–STING Pathway Enhances Immunomodulation by 25HC‐Activated MDSCs

**FIGURE 4:**
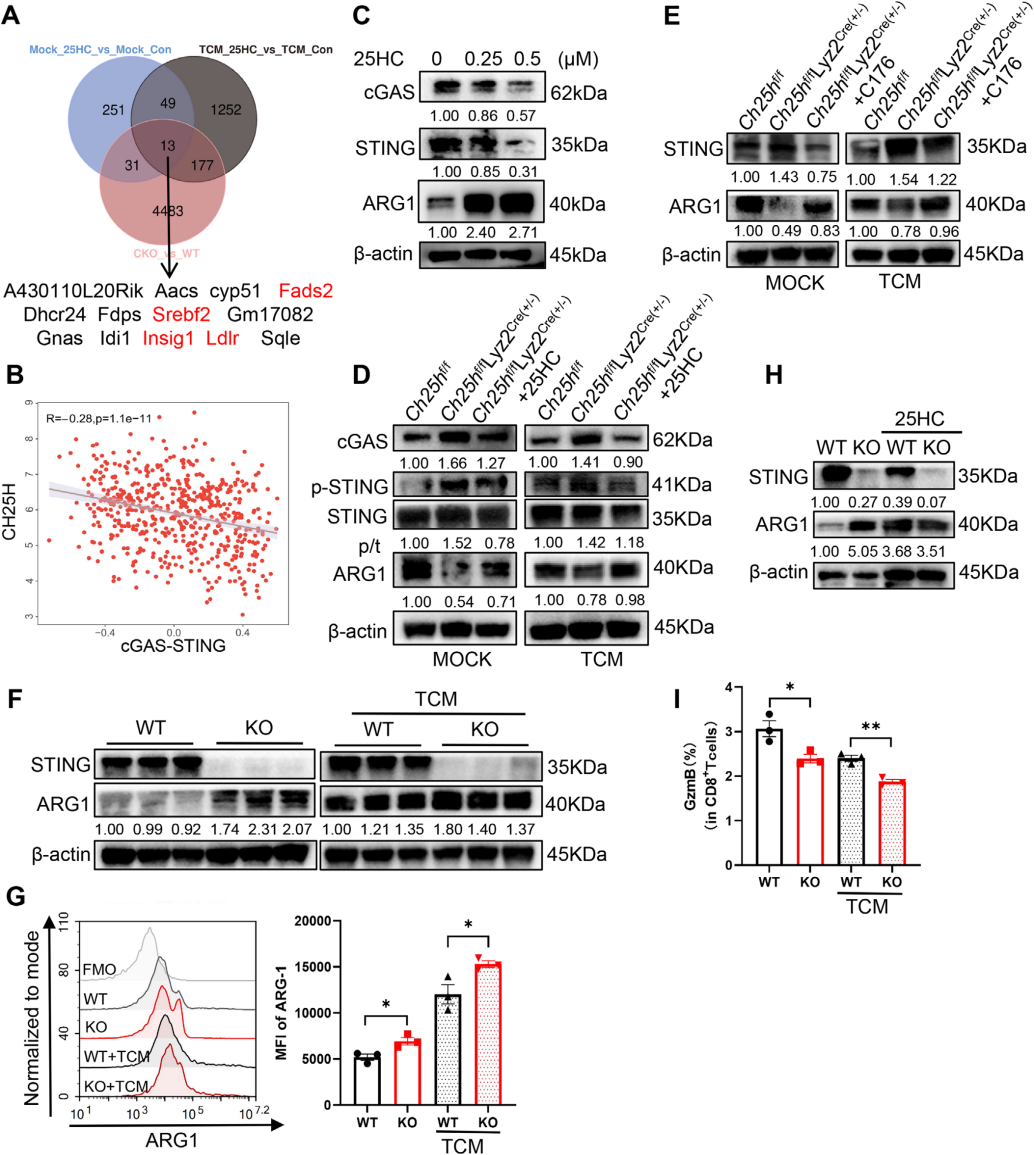
Regulation of MDSCs by 25HC is dependent on the cGAS–STING pathway. (A) Venn plot analysis of RNA transcript sequencing results of WT MDSCs treated with 25HC, either alone or in combination with MC38‐TCM, as well as *Ch25h^f/f^* Lyz2^Cre(±)^ MDSCs (with *Ch25h^f/f^* MDSCs serving as the control). Acas: acetoacetyl‐coenzyme A synthetase; Cyp51: cytochrome P450 family 51; Dhcr24: 24‐dehydrocholesterol reductase; Fdps: farnesyl diphosphate synthase; Gnas: guanine nucleotide‐binding protein, alpha stimulating; Idi1: isopentenyl‐diphosphate delta isomerase 1; Sqle: squalene epoxidase. (B) cGAS–STING scores were calculated by aggregating gene sets from the GOBP_CGAS_STING_SIGNALING_PATHWAY.v2024.1.Hs pathway in the MSigDB database (https://www.gsea‐msigdb.org/gsea/msigdb) using GSVA. The CH25H and cGAS–STING scores were analyzed using Spearman correlation analysis and visualized with scatter plots. (C) Changes in cGAS and STING, as well as ARG1 protein levels in WT MDSCs, were detected via Western blotting (WB) following 25HC treatment (using DMSO as the control). (D) The phosphorylation activation level of STING and the protein level changes of cGAS and ARG1 were assessed after CH25H‐specific knockdown and subsequent treatment with 25HC in MDSCs from both the mock group (induced by RPMI1640 medium) and the MC38‐TCM treatment group (using *Ch25h^f/f^* MDSCs as the control). p/t: phosphorylated protein/total protein. (E) The changes in ARG1 expression in *Ch25h^f/f^* Lyz2^Cre(±)^ MDSCs (using *Ch25h^f/f^* MDSCs as the control) were evaluated after treatment with C176 (1 µM; using the same volume of DMSO as the control condition) for 6 h. (F, G) WB and flow cytometry were employed to detect ARG1 expression in MDSCs from *Tmem173*‐KO mice (using WT MDSCs as the control). (H) WB analysis of ARG1 expression levels in *Tmem173*‐KO MDSCs (using WT MDSCs as the control) induced by the combination of 25HC and MC38‐TCM treatment. (I) A functional inhibition assay was conducted to assess T cell activity after 48 h of coculture of *Tmem173*‐KO MDSCs with CD8^+^ T cells, using WT MDSCs as the control. Comparisons with controls are indicated, where ns denotes no significant difference, *p < 0.05, **p < 0.01, and ***p < 0.001.

To elucidate the mechanisms by which CH25H‐derived 25HC accumulation enhance the immunosuppressive capacity of tumor‐infiltriting MDSCs, we conducted RNA transcriptome sequencing on BM‐derived MDSCs (BM‐MDSCs) that were exogenously supplied with 25HC, alongside a control group. Additionally, we performed RNA sequencing (RNA‐seq) on BM‐MDSCs derived from *Ch25h* ^f/f^ Lyz2^Cre^ mice and their *Ch25h* ^f/f^ counterparts. Venn diagram analysis revealed that 13 genes were commonly differentially expressed; these genes were downregulated following 25HC stimulation and upregulated following CH25H medulla‐specific knockdown. Notably, four of these 13 common differential genes exhibited a positive correlation with the stimulator of interferon genes (STING) activation, including fatty acid desaturase 2 (*Fads2*) [22], low density lipoprotein receptor (*Ldlr*) [23, 24], sterol regulatory element binding protein 2 (*Srebf2*) [25] and insulin‐induced gene 1 (*Insig1*) [26] (Figure 4A). In addition, it has been reported that 25HC protects against cerebral ischemia–reperfusion injury by inhibiting STING activity [27], and that STING activation is negatively correlated with ARG1 expression [28]. Our correlation analysis indicated that cyclic guanosine monophosphate (GMP)–AMP synthase (cGAS)–STING pathway scores were inversely related to CH25H levels in CRC tissues. Therefore, we directed our attention to the classical cGAS–STING signaling pathway (Figure 4B). Consistent with our hypothesis, ARG1 expression was significantly elevated in WT BM‐MDSCs treated with varying concentrations of 25HC, while the protein levels of both cGAS and STING exhibited a concentration‐dependent decrease (Figure 4C). Additionally, assays conducted on *Ch25h* ^f/f^Lyz2^Cre(±)^ MDSCs confirmed that cGAS protein levels were upregulated and STING was activated following CH25H reduction in MDSCs. These effects were diminished upon complementary stimulation with 25HC (Figure 4D). A similar pattern was observed in MDSCs treated with MC38‐TCM (Figure 4D). Thus, we propose that the effect of 25HC, metabolized by CH25H, on promoting ARG1 expression in MDSCs may be mediated through the cGAS–STING pathway.

Based on the aforementioned findings, we utilized C176, a potent and selective STING inhibitor [29, 30], to treat *Ch25h* ^f/f^Lyz2^Cre(±)^ MDSCs. Notably, in contrast to the downregulation of ARG1 observed following CH25H knockdown, treatment with C176 resulted in a reversal of this effect. Furthermore, these alterations were consistent with the effects of 25HC treatment (Figure 4E). Subsequently, we performed a similar assay on *Tmem173* (the genetic name of STING)‐knockout (KO) mice after isolating BM‐MDSCs and noted a significant increase in ARG1 protein levels compared to WT MDSCs. This increase was evident in both the normal medium‐treated and the MC38‐TCM treated groups (Figure 4F,G). Additionally, we treated both WT and *Tmem173*‐KO MDSCs with 25HC and MC38‐TCM, observing no significant changes and, in some cases, a slight downregulation of ARG1 protein levels following 25HC stimulation post‐*Tmem173* KO (Figure 4H). This finding suggests that 25HC produced by CH25H metabolizing cholesterol upregulates ARG1 expression in MDSCs through a STING‐dependent pathway. Moreover, *Tmem173*‐KO MDSCs displayed a notable decrease in GzmB levels compared to WT MDSCs (Figure 4I), indicating a more substantial inhibition of cocultured anti‐CD3 and anti‐CD28‐induced CD8^+^ T cell activation. In addition, we detected cytokines and chemokines downstream of the cGAS–STING pathway using qPCR and enzyme‐linked immunosorbent assay (ELISA), known to induce CD8^+^ T‐cell activation, such as IFN‐β and C–C motif chemokine ligand 5 (CCL5). However, the results indicated no significant differential changes in the levels of these cytokines in either the WT MDSCs or the *Ch25h* ^f/f^Lyz2^Cre(±)^ MDSCs (Figure S4A,B). Collectively, these observations imply that 25HC activates MDSCs and enhances their immunosuppressive function by inhibiting the cGAS–STING pathway.

### 2.5 CH25H Enhances ARG1 Expression by Suppressing TBK1–RIPK3 Signaling in MDSCs

**FIGURE 5:**
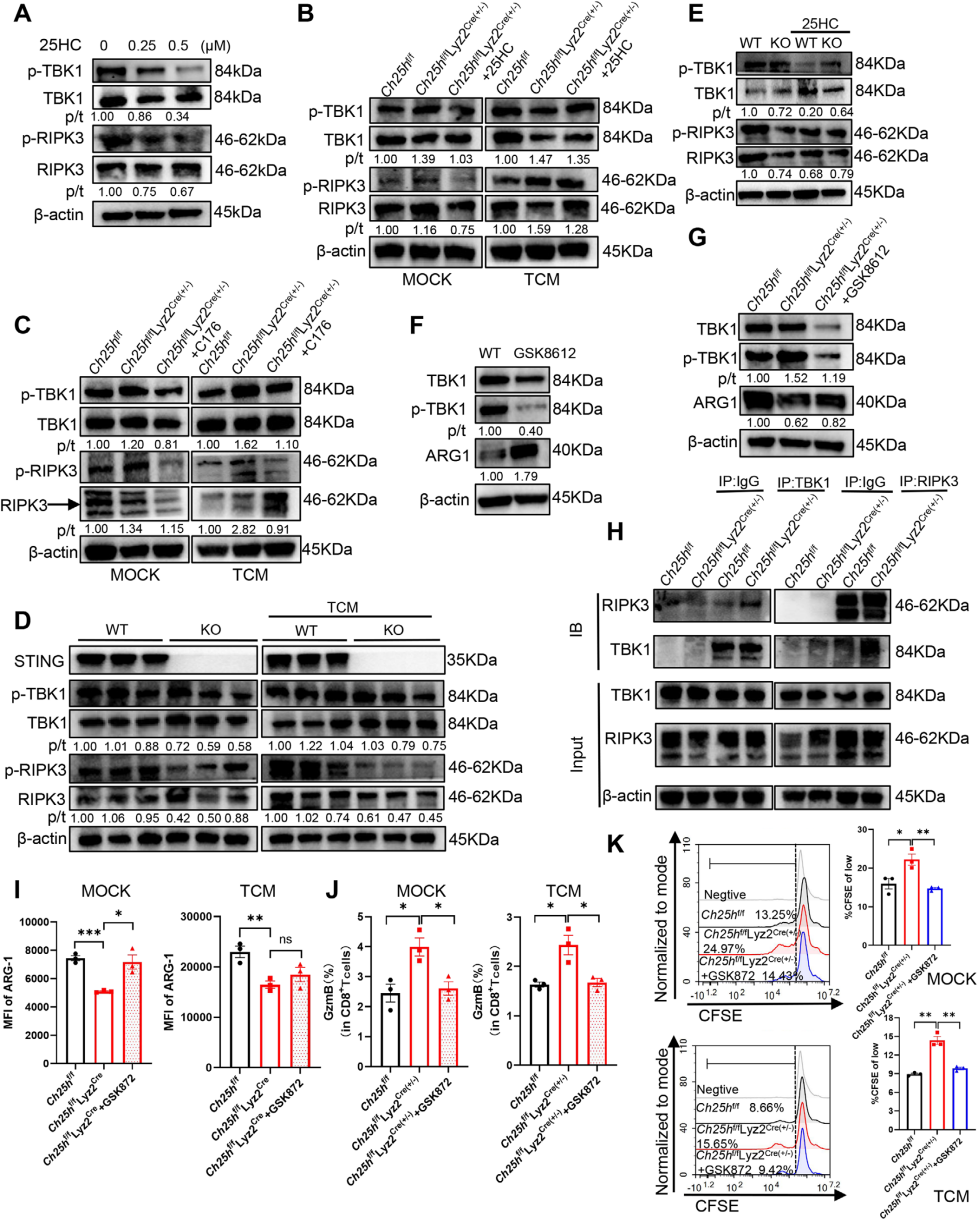
CH25H deletion inhibits ARG1 expression by activating the TBK1–RIPK3 complex. (A) WB analysis shows the phosphorylation levels and total protein levels of TBK1 and RIPK3 in WT MDSCs treated with 25HC (using DMSO as the control condition). (B) The activation levels of TBK1 and RIPK3 phosphorylation, as well as their total levels, are assessed following CH25H‐specific knockdown and subsequent treatment with 25HC in MDSCs from both the mock group and the MC38‐TCM treatment group (using *Ch25h^f/f^* MDSCs as the control). (C) Similar assessments of TBK1 and RIPK3 phosphorylation and total levels are conducted after CH25H‐specific knockdown and C176 stimulation in MDSCs from the mock and MC38‐TCM treatment groups (using *Ch25h^f/f^* MDSCs as the control). (D) The total and phosphorylated expression levels of TBK1 and RIPK3 are compared between WT and *Tmem173*‐KO MDSCs, using WT MDSCs as the control. (E) WB analysis reveals the protein phosphorylation activation levels and total expression of TBK1 and RIPK3 in *Tmem173*‐KO MDSCs induced by the combination of 25HC and MC38‐TCM treatment, using WT MDSCs as the control. (F) WB demonstrates the phosphorylation levels and total protein levels of TBK1, alongside ARG1 expression in WT MDSCs treated with GSK8612 at a concentration of 10 µM for 24 h (using DMSO as the control condition). (G) The alterations in ARG1 and TBK1 expression in *Ch25h^f/f^* Lyz2^Cre(±)^ MDSCs (using *Ch25h^f/f^* MDSCs as the control) following a similar treatment with GSK8612 (10 µM) for 24 h. (H) CoIP assays demonstrate the interactions between TBK1 and RIPK3 proteins in *Ch25h^f/f^* Lyz2^Cre(±)^ MDSCs compared to *Ch25h^f/f^* MDSCs. (I) Flow cytometry assesses ARG1 expression in *Ch25h^f/f^* Lyz2^Cre(±)^ MDSCs, with *Ch25h^f/f^* MDSCs serving as a control, after 24 h of treatment with GSK872 (20 µM), either alone or in combination with MC38‐TCM (*n* = 3). (J) Flow cytometry assays evaluate the functional inhibition of CD8^+^ T cells following 48 h of coculture with MDSCs treated with GSK872, with or without MC38‐TCM (using *Ch25h^f/f^* MDSCs as the control; *n* = 3). (K) Flow cytometry assays assess the inhibitory effects on CD8^+^ T cell proliferation after 72 h of coculture with MDSCs treated with GSK872, both in the presence and absence of MC38‐TCM (using *Ch25h^f/f^* MDSCs as the control; *n* = 3). Statistical comparisons are made against controls, with ns indicating no significant difference, *p < 0.05, **p < 0.01, and ***p < 0.001.

Previous studies have elucidated that STING, an ER membrane protein, translocates to the Golgi apparatus from the ER to activate tank‐binding kinase 1 (TBK1) upon receiving 2′,3′‐cyclic‐GMP‐AMP (cGAMP) signals [31]. Consequently, we investigated whether the STING downstream product TBK1 was also inhibited by CH25H and 25HC. In alignment with our expectations, the Western blot assay revealed a sequential decrease in the ratio of phosphorylated TBK1 to total TBK1 levels following stimulation with varying concentrations of 25HC (Figure 5A). This finding indicated that 25HC enhances the expression of ARG1 by inhibiting the phosphorylation of TBK1 in a concentration‐dependent manner. Similarly, we observed increased phosphorylation of TBK1 in *Ch25h* ^f/f^Lyz2^Cre(±)^ MDSCs compared to *Ch25h* ^f/f^ MDSCs (Figure 5B,C). However, this enhancement was attenuated by STING inhibition following the addition of either 25HC or C176, as well as during treatment with MC38‐TCM (Figure 5B,C). Furthermore, to strengthen our hypothesis, we noted a significant reduction in the ratio of phosphorylated TBK1 to total TBK1 levels in *Tmem173*‐KO MDSCs compared to WT MDSCs, regardless of MC38‐TCM treatment (Figure 5D). Following the addition of 25HC to *Tmem173*‐KO MDSCs, the activation of TBK1 did not exhibit significant changes compared to *Tmem173*‐KO MDSCs without 25HC treatment (Figure 5E). Furthermore, we utilized GSK8612, a selective inhibitor of TBK1, in conjunction with MC38‐TCM. We observed that the expression of ARG1 significantly increased following the inhibition of TBK1 phosphorylation induced by the treatment of WT MDSCs (Figure 5F). Consistent with our previous findings, the treatment of *Ch25h* ^f/f^Lyz2^Cre(±)^ MDSCs with GSK8612 also led to a reduction in the degree of TBK1 phosphorylation, which in turn promoted an increase in ARG1 expression (Figure 5G). Taken together, the above results further support the notion that 25HC promotes ARG1 expression, mediating the immunosuppressive function of MDSCs through the STING‐TBK1 pathway.

TBK1 is expressed across all tissues and functions as a nonclassical IκB kinase (IKK) [32]. Molecular interaction network analysis indicates that TBK1 may interact with receptor‐interacting protein kinase 3 (RIPK3; Figure S5A,B), which our previous studies identified as an inhibitor of ARG1 expression in MDSCs [9, 10]. Furthermore, TBK1 has been reported to mediate the phosphorylation of RIPK3, thus activating the protein [33]. Based on this, we hypothesized that CH25H‐25HC suppression of TBK1 would enhance ARG1 expression by reducing RIPK3 activity. Following stimulation with varying concentrations of 25HC, we observed a concentration‐dependent decrease in the ratio of phosphorylated RIPK3 to total RIPK3, mirroring the pattern seen for TBK1 (Figure 5A). As anticipated, RIPK3 protein phosphorylation levels were elevated in *Ch25h* ^f/f^Lyz2^Cre(±)^ MDSCs compared to *Ch25h* ^f/f^MDSCs; however, this upregulation diminished with the addition of either 25HC or C176, similar to the response in MDSCs induced by MC38‐TCM (Figure 5B,C). Consistent with the behavior of TBK1, RIPK3 activation was also reduced in *Tmem173*‐KO MDSCs compared to WT MDSCs, and this suppression persisted with the addition of 25HC backfill treatment, aligning with the control group (Figure 5D,E). Additionally, co‐IP experiments further supported the interaction between TBK1 and RIPK3 in MDSCs. Notably, the protein interactions between these two proteins were significantly enhanced in *Ch25h* ^f/f^Lyz2^Cre(±)^ MDSCs compared to *Ch25h* ^f/f^ MDSCs (Figure 5H), suggesting that CH25H knockdown in MDSCs promotes the STING and its downstream TBK1–RIPK3 signaling axis.

To investigate whether CH25H‐enhanced ARG1 expression was dependent on the inhibition of RIPK3, we treated *Ch25h* ^f/f^Lyz2^Cre(±)^ MDSCs with the RIPK3‐specific inhibitor GSK872 [9, 10] to block RIPK3 upregulation. The results indicated that the administration of GSK872, regardless of the presence or absence of MC38‐TCM treatment, led to an upregulation of ARG1 expression in *Ch25h* ^f/f^Lyz2^Cre(±)^ MDSCs (Figure 5I). Additionally, there was a significant reversal of the increased GzmB expression and proliferative capacity observed in CD8^+^ T cells (Figure 5J,K).

In conclusion, the data suggest that the absence of CH25H in MDSCs activates the STING pathway and suppresses ARG1 expression by facilitating the formation of the TBK1–RIPK3 complex. CH25H‐derived 25HC is implicated in the pathogenesis of CRC by inhibiting the STING–TBK1–RIPK3 signaling axis and promoting the expression of the immunosuppressive molecule ARG1, which enhances the immunosuppressive effects of CRC‐associated MDSCs.

### 2.6 25HC Promotes Tumor Progression by Enhancing the Immunoregulatory Activity of MDSCs

**FIGURE 6:**
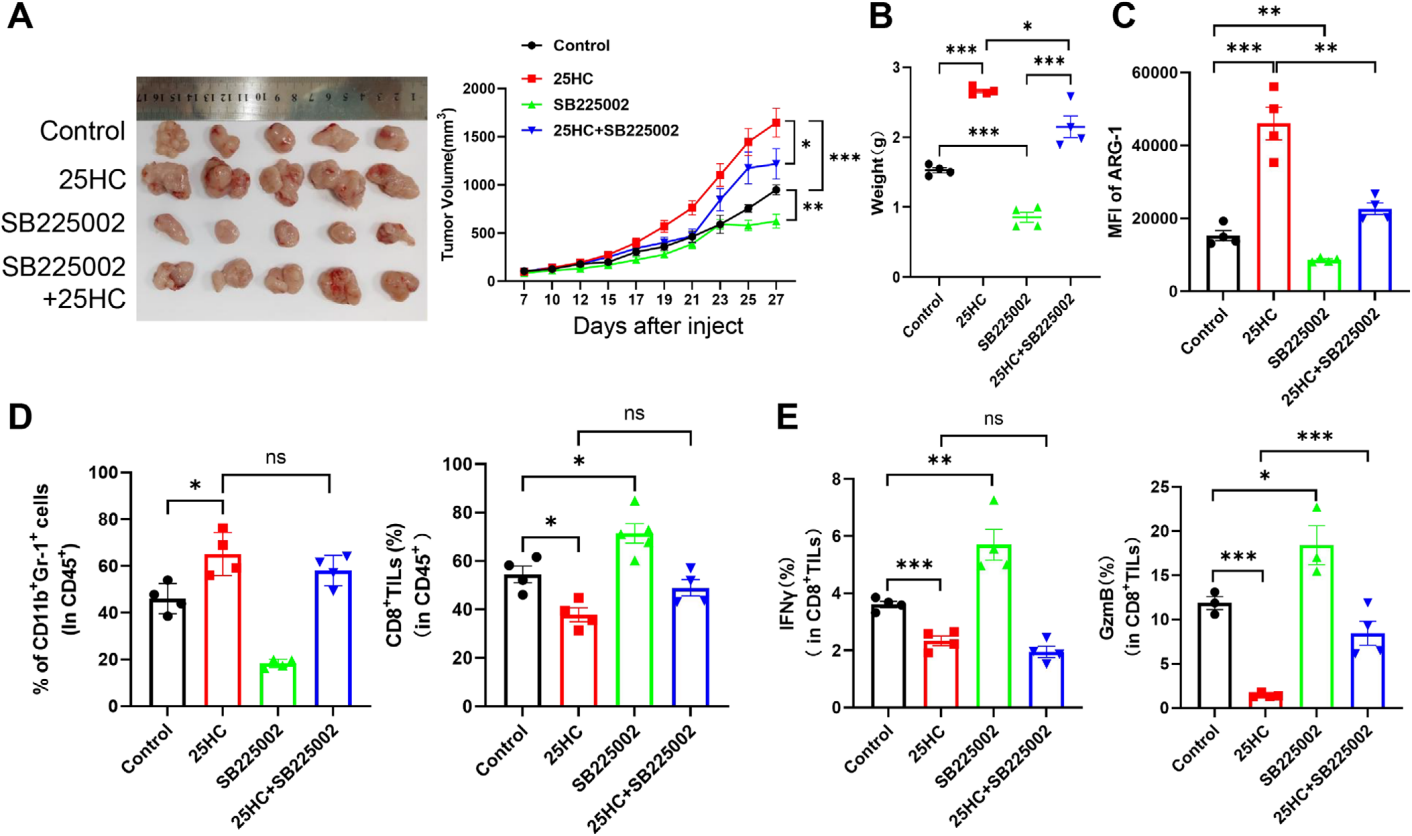
Functional reprogramming of tumor‐associated MDSCs by 25HC. (A) and (B) Physical images of tumors, along with dynamic growth curves and mass measurements in subcutaneously implanted MC38‐loaded mice following injection of 25HC, both combined and uncombined with SB225002 (*n* = 5). (C) The levels of ARG1 within the tumor MDSCs of mice in each group. (D) The percentage of MDSCs and CD8^+^ TILs in the TME (*n* = 4). (E) The percentage of IFNγ and GzmB in CD8^+^ TILs derived from CD45^+^ cells (*n* = 4). Statistical comparisons indicate ns for no significant difference, *p < 0.05, **p < 0.01, and ***p < 0.001.

To further elucidate the pivotal role of CH25H‐derived 25HC in the context of CRC‐associated MDSCs, we developed an MC38‐loaded tumor model in WT mice. We administered 25HC alongside the MDSC inhibitor SB225002, a potent and selective C–X–C motif chemokine receptor 2 (CXCR2) antagonist that diminishes MDSC chemotaxis by inhibiting their accumulation [9], until the mice were euthanized. Compared to the control group, the administration of 25HC significantly promoted the growth of MC38 subcutaneous tumors, as evidenced by an accelerated tumor growth rate and increased tumor volumes and weights. Conversely, the administration of SB225002 inhibited tumor progression (Figure 6A,B). Additionally, we assessed the proportion of tumor‐infiltrating immune cells and their functional activity. The expression of ARG1 in MDSCs was significantly elevated in tumor‐bearing mice treated with 25HC compared to the no‐treatment group. However, this increase was reversed following coadministration of SB225002, suggesting that the depletion of MDSCs notably mitigates the tumor‐promoting effects of 25HC (Figure 6C). Furthermore, the percentage of tumor‐associated MDSCs in the 25HC treatment group was substantially higher than that in the control group, while the infiltration rate of total tumor‐infiltrating CD8^+^ T‐cells (CD8^+^ TILs) was reduced relative to the control group (Figure 6D). Concurrently, the expression levels of IFNγ and GzmB in CD8^+^ TILs were also significantly suppressed (Figure 6E). These findings indicate that 25HC enhances the immunosuppressive activity of tumor‐associated MDSCs, thereby promoting tumor immune escape.

### 2.7 Administration of 25HC or C176 Reverses CH25H Myeloid‐Specific Knockdown Effects on Tumor Progression

**FIGURE 7:**
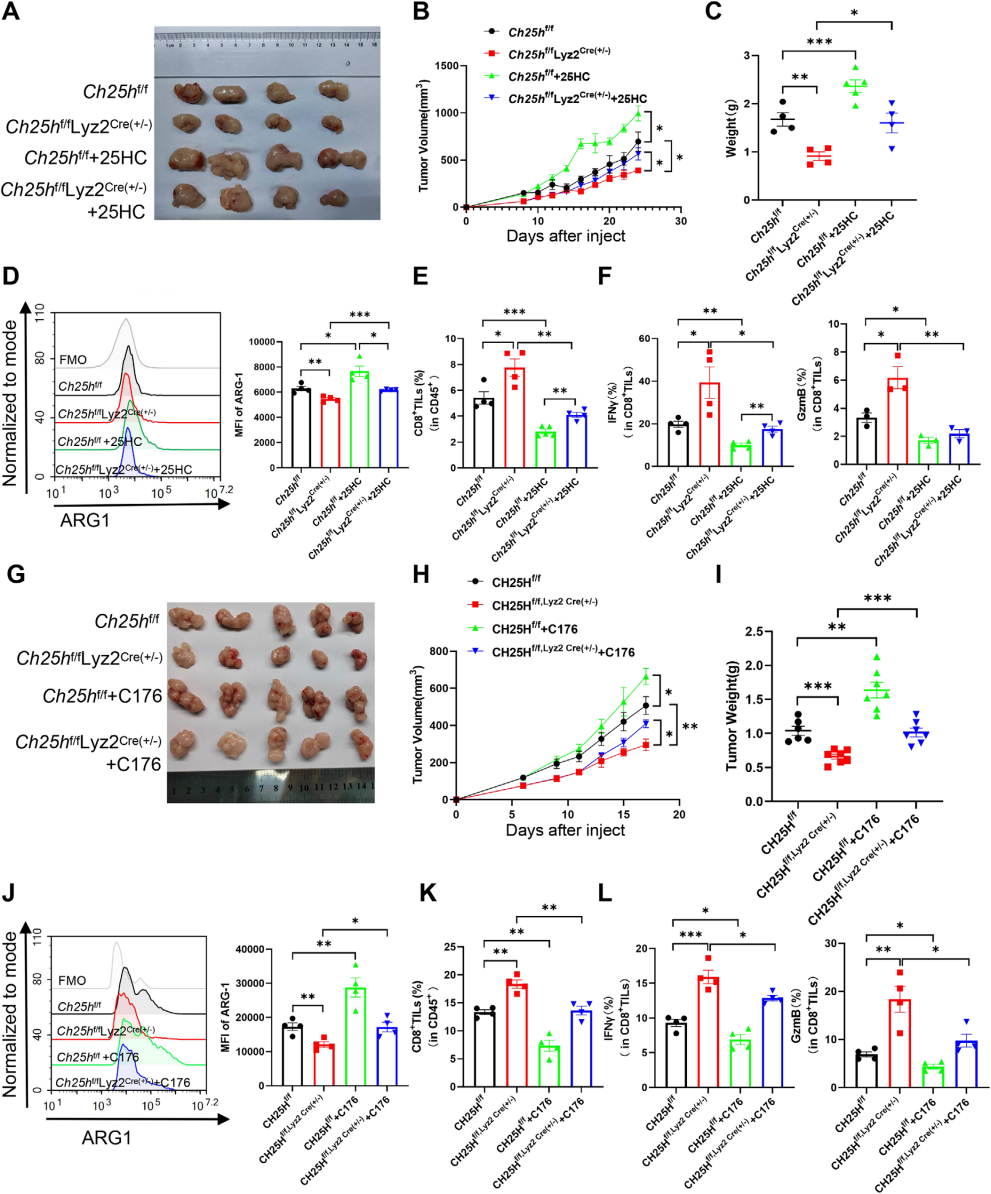
CH25H‐deficient MDSCs can reverse their antitumor effects following stimulation with 25HC and C176. Panels A–F present physical images of solid tumor volume (A), tumor dynamic growth curves (B), and mass (C), as well as ARG1 expression in tumor MDSCs (D), the percentage of CD8^+^ TILs in CD45^+^ cells (E), and the percentage of IFNγ^+^ or GzmB^+^ CD8^+^ TILs (F) in *Ch25h^f/f^* Lyz2^Cre(±)^ mice treated with or without 25HC (*n* = 4). Panels G–L depict physical drawings of solid tumor volume (G), tumor dynamic growth curves (H), and mass (I), along with ARG1 expression in tumor MDSCs (J), the percentage of CD8^+^ TILs in CD45^+^ cells (K), and the percentage of IFNγ^+^ or GzmB^+^ CD8^+^ TILs (L) in *Ch25h^f/f^* Lyz2^Cre(±)^ mice treated with or without C176 (*n* = 5). Statistical comparisons indicate no significant difference (ns) compared to controls, with significance levels of *p < 0.05, **p < 0.01, and ***p < 0.001.

We next conducted a series of in vivo validation experiments utilizing CH25H myeloid‐specific knockdown mice. Specifically, 6–8‐week‐old male *Ch25h* ^f/f^Lyz2^Cre(±)^ mice and their corresponding CH25H^f/f^ control mice were subcutaneously inoculated with MC38 cells. After 8 days, the mice were randomly and equitably divided into two groups, which subsequently received daily injections of either 25HC or its vehicle. Compared to the *Ch25h* ^f/f^ mice, the subcutaneous tumors in the *Ch25h* ^f/f^Lyz2^Cre(±)^ mice demonstrated a slower growth rate, along with reduced tumor volume and weight following the myeloid‐specific knockdown of CH25H. Conversely, the administration of 25HC to counteract the absence of its effects resulted in accelerated tumor growth, yielding tumor volume and weight comparable to those observed in the *Ch25h* ^f/f^ tumor‐bearing mice (Figure 7A–C). Further analysis of immune‐infiltrating cells within the TME indicated that ARG1 expression was lower in tumor‐associated MDSCs from *Ch25h* ^f/f^Lyz2^Cre(±)^ homozygous mice compared to those from *Ch25h* ^f/f^ homozygous mice. However, the reintroduction of 25HC led to an increase in ARG1 expression (Figure 7D). Additionally, the percentage of CD8^+^ TILs and the expression of IFNγ or GzmB were significantly higher in *Ch25h* ^f/f^Lyz2^Cre(±)^ mice than in CH25H^f/f^ mice, although both were diminished by the administration of 25HC treatment (Figure 7E,F). Consistent with previous findings suggesting that 25HC promotes tumor progression (Figure 6A–E), we observed continued tumor progression in *Ch25h* ^f/f^ tumor‐bearing mice that received 25HC.

We also administered C176 to tumor‐bearing CH25H myeloid‐specific knockdown mice, which were randomly assigned to groups in accordance with our previous protocols. Consistent with the in vivo results observed following 25HC injection, tumor growth in *Ch25h* ^f/f^ ruffled mice was significantly accelerated after the administration of C176. This acceleration led to an increase in both tumor volume and weight, alongside a notable enhancement in ARG1 expression within tumor‐infiltrating MDSCs. Conversely, the proportion of CD8^+^ T cell infiltration, as well as the levels of IFNγ^+^ and GzmB^+^CD8^+^ T cells, were suppressed. Notably, treatment of *Ch25h* ^f/f^Lyz2^Cre(±)^ mice injected with C176 resulted in a marked suppression of the antitumor effect, further accelerating tumor progression, increasing ARG1 expression, and leading to a significant reduction in CD8^+^ TILs and IFNγ^+^ or GzmB^+^ CD8^+^ TILs compared to those injected with the vector control (Figure 7G–L). Collectively, these findings indicate that myeloid‐specific knockdown of CH25H blunts tumor growth; however, the reintroduction of 25HC or the administration of the STING inhibitor C176 can reverse this effect, thereby accelerating tumorigenesis and progression.

In the past 3 years, we collected surgical frozen tumor tissue samples from patients with CRC at various stages of clinical progression and performed serial sectioning. Analysis of CH25H and ARG1 IF staining on these serial sections revealed a significant increase in the infiltration of MDSCs within CRC tumor tissues as tumor progression advanced (Figure S5A,B). More importantly, consistent with the results from the mouse model, colocalization analysis explicitly demonstrated that the expression levels of CH25H (Figure S5A) and ARG1 (Figure S5B) in the infiltrated MDSCs also increased in tandem. These findings not only reinforce our previous conclusions but also provide a crucial foundation for further exploration of the clinical significance of targeting MDSCs in CRC treatment.

## 3 Discussion

In this study, we observed that both CH25H and its downstream metabolite 25HC were upregulated in tumor‐associated MDSCs, which in turn suppressed the cGAS–STING pathway. This suppression led to the downregulation of the inhibitory effects of the downstream TBK1–RIPK3 complex on ARG1, thus enhanced the immunosuppressive function of MDSCs, facilitating immune escape in CRC. Conversely, the specific deletion of CH25H in MDSCs resulted in a significant loss of immunosuppressive function and markedly enhanced CD8^+^ T cell infiltration and antitumor activity. These findings indicate that CH25H plays a vital role in regulating the functional phenotype of tumor‐associated MDSCs and represents a crucial target for reversing tumor immune evasion through lipid metabolic reprogramming in MDSCs.

CRC is a malignant tumor characterized by high morbidity and mortality rates worldwide, representing a significant threat to public health due to the considerable challenges associated with its treatment, particularly in the metastatic stage. Recent advancements in the use of immune checkpoint inhibitors (ICIs), such as PD‐1/PD‐L1 antibodies, have generated considerable interest in their application for CRC, following their successful use in various tumors, including non—small‐cell lung cancer [34], and melanoma [35]. However, current clinical studies and guidelines indicate that PD‐1/PD‐L1 monotherapy is generally unsuitable for the majority of patients with metastatic CRC. The clinical efficacy of PD‐1/PD‐L1 inhibitors in metastatic CRC is highly contingent upon the microsatellite instability (MSI) and MMR status of the tumor [36, 37, 38]. Numerous clinical trials have demonstrated that PD‐1 inhibitors, including nivolumab and pembrolizumab, exhibit significant efficacy primarily in subpopulations of CRCs characterized by high microsatellite instability (MSI‐H) or defective mismatch repair (dMMR) [36, 39, 40, 41]. Unfortunately, these patients constitute only approximately 4%–5% of the total CRC population. For the majority of metastatic CRC patients with microsatellite‐stable (MSS) or mismatch repair‐proficient (pMMR) phenotypes, the objective response rate (ORR) to single‐agent PD‐1/PD‐L1 inhibitor therapy is exceedingly low, yielding minimal clinically beneficial effects [37, 42, 43]. Consequently, this treatment is not recommended as a standard therapy. Furthermore, patients with MSS CRC often present a highly immunosuppressive microenvironment, characterized by low tumor mutational burden (TMB), a scarcity of tumor‐infiltrating lymphocytes (TILs), and reduced PD‐L1 expression, which collectively hinder the effectiveness of PD‐1/PD‐L1 inhibitors [37, 44, 45]. Moreover, MDSCs represent a prominent cell population that sustains the immunosuppressive microenvironment of CRC [43]. Therefore, targeting MDSCs to enhance immunotherapy outcomes in MSS/pMMR CRC patients emerges as a promising strategy.

CH25H is a conserved IFN‐stimulated gene (ISG) situated on the ER. It plays a crucial role in regulating immune cell function and lipid metabolism by catalyzing the oxidation of cholesterol into 25HC [46]. Recent studies have highlighted significant interactions between CH25H/25HC and the inflammatory response. For instance, lipopolysaccharide (LPS) induces the upregulation of CH25H in both mouse and human macrophages [47,48], which may be pivotal in the pathogenesis of inflammatory bowel disease (IBD), a leading cause of CRC [46]. Notably, 25HC exhibits both proinflammatory and anti‐inflammatory effects, with these variations likely influenced by the concentration of administration and the specific microenvironment, resulting in diverse impacts on gene expression, cell proliferation, differentiation, and apoptosis [49]. Evidence indicates that macrophage‐derived 25HC can enhance the inflammatory response in lipid‐loaded macrophages by modifying cholesterol reservoirs within the plasma membrane. This alteration subsequently affects toll‐like receptor 4 (TLR4) signaling, promoting NF‐κB‐mediated proinflammatory gene expression, which contributes to vascular inflammation and the progression of atherosclerosis [50]. Conversely, krüppel‐like factor 4 (KLF4) has been shown to upregulate Ch25h and liver X receptor (LXR) expression. This KLF4–Ch25h/LXR homeostasis axis exhibits anti‐inflammatory properties and reduces susceptibility to atherosclerosis by inhibiting inflammatory vesicle activity in endothelial cells (ECs) and facilitating a shift from the M1 to M2 phenotype in macrophages [51]. However, during the exploratory phase of our experiments, we observed that the administration of high doses (50 mg/kg) of 25HC intraperitoneally to WT C57BL/6 male rats resulted in limited and progressively slowed tumor growth. Thus, regulating 25HC levels ad libitum may represent a promising strategy for tumor prevention and treatment.

CH25H is prominently expressed in various immune cells, including T‐lymphocytes, macrophages, and dendritic cells (DCs), under physiological conditions, where it plays distinct roles in tumor immunity [52]. Lu et al. demonstrated that tumor‐derived factor (TDF) induces the transcription factor activating transcription factor 3 (ATF3), which subsequently inhibits the expression of CH25H in intratumoral cytotoxic T lymphocytes (CTLs) and stimulates their cellular autophagy. This process ultimately attenuates antitumor immunity and promotes tumor growth. Conversely, the restoration of CH25H expression through pharmacological agents can reverse this effect and enhance the efficacy of immunotherapy [53]. Xiao et al. reported that IL‐4 and IL‐13 induce CH25H expression in macrophages through the transcription factor STAT6 [16]. The accumulation of 25HC in the lysosomes of macrophages competes with cholesterol for binding to GPR155, thereby inhibiting the mechanistic target of rapamycin complex 1 (mTORC1) kinase. This process activates AMPKα and induces metabolic reprogramming, as well as the phosphorylation of STAT6 at Ser564, which enhances ARG1 production [16]. The observation that CH25H promotes ARG1 expression in myeloid cells aligns with our findings. Lu et al. also discovered that CH25H deficiency in DCs facilitates the fusion of endo‐phagosomes and lysosomes, accelerates lysosomal degradation, and restricts the cross‐presentation of tumor antigens in intratumoral DCs. This results in accelerated tumor growth, reduced infiltration, and impaired activation of intratumoral CD8^+^ T cells [54]. In our study, the downregulation of CH25H within MDSCs inhibits ARG1 expression by activating the STING–TBK1–RIPK3 signaling axis, thereby enhancing the antitumor effect. Therefore, CH25H may serve as a crucial metabolic target for immunotherapy, potentially providing novel insights for subsequent tumor‐targeted drug development.

Two distinct types of cGAS–STING signaling pathways have been identified: the classical and nonclassical pathways [55, 56]. The classical pathway is primarily characterized by the expression of cytokines, including type I interferons, along with other cytokines [57]. In contrast, nonclassical pathways play a crucial role in regulating various conductance modalities. One notable pathway involves the retention of STING in the ER in a resting state through its binding to lipid raft proteins, including stromal interaction molecule 1 (STIM1) [58]. However, there are currently no reports detailing the effects of CH25H/25HC on cGAS or cGAMP. Our study demonstrated that CH25H deficiency markedly enhanced STING‐TBK1 activity, a phenomenon that was counteracted by the administration of 25HC in MDSCs. Importantly, the inhibition of STING reversed the tumor reduction observed in mice with myeloid CH25H knockdown. While the upregulation of CH25H and its metabolite 25HC in tumor‐associated MDSCs has been shown to suppress STING signaling, the precise molecular mechanisms driving this effect require further investigation and validation. Nonetheless, it has been documented that the accumulation of 25HC in cells induces ER stress [59, 60], which is responsible for the activation of MDSCs [12]. Furthermore, it has been observed that protein kinase RNA‐like endoplasmic reticulum kinase (PERK), a molecule associated with the unfolded protein response (UPR) that is highly expressed in tumor‐infiltrating MDSCs, can inhibit STING‐mediated type I interferon responses and enhance its immunosuppressive function by maintaining mitochondrial homeostasis [61]. Therefore, the CH25H cholesterol metabolite 25HC may promote the immunosuppressive effects of MDSCs by inducing ER stress and upregulating the expression levels of PERK, thereby inhibiting STING and its downstream‐mediated type I interferon response. In our exploration of this mechanism, we observed that while STING was suppressed by CH25H/25HC, neither ER stress nor the activation of PERK was induced by CH25H/25HC in MDSCs. This finding suggests that CH25H/25HC inhibits STING signaling through an alternative mechanism in MDSCs.

Recent research indicates that the accumulation of 25HC enhances the binding of 3‐hydroxy‐3‐methylglutaryl coenzyme A reductase (HMGCR) to insig proteins located on the ER. This interaction can activate and initiate the endoplasmic reticulum‐associated degradation (ERAD) mechanism, thereby contributing to the ubiquitin‐mediated degradation of HMGCR [62]. Additionally, other studies have identified the suppressor/enhancer of Lin‐12‐like (SEL1L)–hydroxymethylglutaryl reductase degradation protein 1 (HRD1) complex as a highly conserved component of ERAD, capable of ubiquitinating STING for degradation [63]. Consequently, we hypothesize that 25HC may trigger ERAD in this context, promoting the ubiquitination and degradation of the initial STING molecule located on the ER. This process ultimately results in the upregulation of ARG1 levels, enhancing the immunosuppressive capacity of MDSCs and facilitating tumor immune escape. Our RNA‐seq analysis identified four differential genes—*Fads2*, *Ldlr*, *Srebf2*, and *Insig1*—that were positively correlated with STING activation. These genes exhibited downregulation in response to 25HC and upregulation following CH25H knockdown, suggesting that the regulatory mechanism of STING by CH25H/25HC may be intricately linked to one or more of these molecules.

Nevertheless, this study has several limitations. Notably, *Ch25h* ^f/f^Lyz2^Cre(±)^ mice demonstrated a knockdown effect of CH25H in MDSCs, rather than a complete KO of CH25H. We were unable to obtain pure *Ch25h* ^f/f^Lyz2^Cre^ mice, likely due to the potential adverse effects of a complete KO of this metabolism‐related gene on overall physical condition, which may even be lethal during embryonic development due to natural selection pressures. While we have confirmed that *Ch25h* ^f/f^Lyz2^Cre(±)^ heterozygous knockdown mice exhibit a certain degree of KO efficiency, we aim to further validate the impact of CH25H on the immune function of MDSCs. To achieve this, a viable approach involves inducing Cre recombinase–estrogen receptor T2 fusion protein (Cre–ERT2) through the administration of an appropriate dose of tamoxifen to adult *Ch25h* ^f/f^Lyz2^Cre^ mice, which is anticipated to enhance KO efficiency. However, it is essential to acknowledge that tamoxifen is toxic to mice, thus requiring careful veterinary oversight throughout the procedure.

In conclusion, we have thoroughly examined the regulatory effects and mechanisms of CH25H on the function of CRC‐associated MDSCs. Our findings indicate that CH25H and its metabolite, 25HC, which are elevated in tumor‐infiltrating MDSCs, upregulate the expression of ARG1 and enhance immunosuppressive activity by inhibiting the STING–TBK1–RIPK3 signaling axis. This research provides a promising new strategy for further investigations into the reprogramming of cholesterol metabolism in tumor‐infiltrating MDSCs and its role in modulating tumor immune invasion. Additionally, this work enriches our understanding of the regulatory pathways involved in cholesterol metabolic reprogramming and identifies CH25H as a potential new target for improving the efficacy of immunotherapy in clinical CRC patients.

## 4 Materials and Methods

### 4.1 Patient Samples

Peripheral blood samples were collected from eight patients diagnosed with CRC and five healthy controls at the Chongqing University Cancer Hospital. Blood samples were drawn in the morning, around 8:00 a.m., after a minimum fasting period of 8 h. Subsequently, the fresh blood samples underwent a process for isolating human peripheral blood mononuclear cells (PBMCs). Following gradient centrifugation, the myeloid cells in the middle layer were extracted and analyzed through flow cytometry to assess the levels of target molecules in the MDSCs present in the blood samples. The CD11b^+^ HLA‐DR^−^ phenotype was utilized as a marker for identifying human MDSCs. Additionally, surgical pathological paraffin sections of tumors and adjacent healthy tissue were obtained from eight pairs of CRC patients. Surgical pathology frozen samples were collected from patients with CRC at various stages of the disease. The selection criteria for these samples included male patients aged between 50 and 70 years, with no other concomitant cancers or diseases.

### 4.2 Mice

Male WT C57BL/6J mice, aged 4–8 weeks, were sourced from Vital River (Beijing, China). The *Ch25h* ^floxp/floxp^ (*Ch25h* ^f/f^) mice possess two floxp sites flanking the exon of the *Ch25h* gene. Both *Ch25h* ^f/f^ and Lyz2^Cre^ mice were obtained from the Shanghai Model Organisms Center (Shanghai). Lyz2^Cre^ mice were subsequently crossed with *Ch25h* ^f/f^ mice to generate offspring designated as *Ch25h* ^f/−^Lyz2^Cre^ mice. Further breeding of these offspring resulted in the production of *Ch25h* ^f/f^ and *Ch25h* ^f/f^Lyz2^Cre^ experimental mice. Observations of the resulting offspring from multiple generations indicated that *Ch25h* ^f/f^Lyz2^Cre^ mice exhibited low survival rates and a potential for embryonic lethality. Consequently, all subsequent experiments utilized *Ch25h* ^f/f^Lyz2^Cre(±)^ mice, whose knockdown efficacy was verified. All mice were maintained in a pathogen‐free environment at Chongqing University Cancer Hospital, with six mice housed per cage in filtered cages under standardized conditions (strict 12‐h light–dark cycle from 8:00 a.m. to 8:00 p.m., humidity maintained at 50 ± 15%, and temperature regulated at 22 ± 1°C). Autoclaved food and water were provided ad libitum. For the animal experiments, mice aged 4–8 weeks were randomly assigned to various groups, with littermate controls included in each experimental setup.

Following the outlined procedures, genotyping was conducted by excising a small piece of ear or tail tissue from the mice, followed by genomic deoxyribonucleic acid (DNA) extraction using the Rapid DNA Extraction Detection Kit (TIANcombi DNA Lyse&Det PCR Kit, Catalogue No. KG203). The target gene was subsequently amplified through PCR employing specific primers tailored for the target gene. The PCR reaction was executed in a total volume of 20 µL, which included 2 µL of template DNA, 1 µL of each 10 µM forward and reverse primers, 10 µL of 2 × Taq polymerase, and an appropriate volume of 1 × sterile double‐distilled water. After amplification, agarose gel electrophoresis was performed using a 1% agarose gel containing 10% SuperRed/GelRed nucleic acid dye (Biosharp, Catalogue No. BS354A). A DNA ladder served as a size reference to confirm the presence and size of the amplified target gene.

### 4.3 Cell Culture

The mouse colon adenocarcinoma cell line, designated as MC38 (Catalogue No. 1101MOU‐PUMC000523), was obtained from the National Infrastructure for Cell Line Resources (NICR). Additionally, the mouse CRC cell line CT26 (Catalogue No. ATCC CRL‐2638) was sourced from the American Type Culture Collection (ATCC), while the mouse Lewis lung carcinoma cell line (LLC, Catalogue No. GDC0670) and the mouse melanoma cell line (B16‐F10, Catalogue No. GDC0677) were obtained from the China Center for Type Culture Collection (CCTCC). All cell lines were cultured in Dulbecco's modified Eagle medium (DMEM; Gibco), supplemented with 1 mL of fetal bovine serum (FBS; Gibco) and 100 µL of penicillin–streptomycin (Beyotime, Catalogue No. C0222), resulting in a total volume of 10 mL of complete medium. The cultures were maintained at 37°C in a humidified incubator with 5% CO_2_.

### 4.4 Animal Models

Prior to injection into mice, MC38, CT26, LLC, and B16‐F10 cell lines were digested with trypsin (Beyotime, Catalogue No. C0201) to detach single cells. The cells were then washed twice with phosphate buffered saline (PBS) and resuspended. A total of 5 × 10^5^ cells (for MC38 and CT26) or 1 × 10^6^ cells (for LLC and B16‐F10) were injected subcutaneously into the thigh region of the lower back of the mice. Tumor volume was assessed using calipers and calculated using the formula [(small diameter)^2^ × (large diameter)/2]. Tumor growth was monitored regularly, and intraperitoneal drug injection was initiated when the tumor volume reached 80–100 mm^3^. Mice were euthanized when the tumor volume exceeded 1500 mm^3^ to obtain tumor tissue for subsequent flow cytometry assays. For 25HC treatment, 8 days after the subcutaneous injection of MC38 cells, intraperitoneal injections of 100 µL (5% dimethyl sulfoxide [DMSO; Beyotime, Catalogue No. ST038] + 60% polyethylene glycol 300 [PEG300; Selleck, Catalogue No. S6704] + 35% sterile saline) or control vehicle were administered once daily for 3 days, delivering 10 mg of 25HC (Cayman, Catalogue No. 11097) per mouse, after which the mice were killed after 20 days. For SB225002 treatment, 8 days postsubcutaneous transplantation of MC38 cells, mice received intraperitoneal injections of SB225002 (Selleck, Catalogue No. S7651) at a dosage of 100 µL (20 mg/kg, 5% DMSO + 40% PEG300 + 55% sterile saline) or control vehicle every 2 days, with mice being sacrificed after 20 days. For C176 treatment, following the injection of MC38 cells for 8 days, mice received intraperitoneal injections of C176 (Selleck, Catalogue No. S6575) at a volume of 200 µL (11.5 mg/kg, 5% DMSO + 40% PEG300 + 5% Tween80 [VETEC, Catalogue No. V900507] + 50% sterile saline) or control vector every 2 days, and were euthanized after 20 days.

### 4.5 MDSC Induction in vitro

MDSCs were isolated and induced as previously described [10]. In this study, we euthanized 8‐week‐old WT or transgenic mice and proceeded to remove their bilateral femurs and tibias. The bones were washed with PBS to eliminate muscle tissue, and their ends were clipped to facilitate the rinsing of BM using a sterile syringe. This rinsing process was repeated several times until the bones were clean. The collected BM cells were then centrifuged at 2500 rpm for 5 min, after which the supernatant was discarded. The cells were treated with erythrocyte lysis buffer (Beyotime, Catalogue No. C3702) and incubated at 37°C for 8 min to lyse the red blood cells before terminating the lysis process and performing a second centrifugation. Subsequently, the cells were resuspended in RPMI‐1640 medium, filtered to remove impurities, and counted.

Following this, we utilized an antimouse MDSC isolation kit (CD11b^+^Gr1^+^) from Stemcell (Catalogue No. 19867) to sort the collected BM cells according to the step‐by‐step instructions provided in the manual. Ultimately, the negative liquid separated by the magnetic rack constituted the MDSCs suspension. The freshly harvested MDSCs were prepared for subsequent experiments after a 48‐h incubation in RPMI‐1640 medium supplemented with 10% Gibco serum and GM‐CSF (35 ng/mL, PeproTech, Catalogue No. 315‐03). The inhibitors used in subsequent in vitro experiments were GSK872 (Selleck, Catalogue No. S8465), GSK8612 (Selleck, Catalogue No. S8872), and C176.

### 4.6 CD8^+^ T Cell Isolation, Activation, and Proliferation Assay

Initially, the spleens of C57BL/6 mice were homogenized to create single‐cell suspensions. Subsequently, CD8^+^ T cells were isolated using the Mouse CD8^+^ T Cell Isolation Kit (StemCell, Catalogue No. 19853) following established protocols [10, 11].

For functional assays, MDSCs were cocultured with anti‐CD3 (3 µg/mL, BioLegend, Catalogue No. 100238) and anti‐CD28 (3 µg/mL, BioLegend, Catalogue No. 102116) activated enriched CD8^+^ T cells at a ratio of 1:3, along with interleukin‐2 (IL‐2, 1 µg/mL, PeproTech, Catalogue No. 212‐12) for 48 h in 96‐well plates. Subsequently, the cells were harvested and stimulated with a Cell Stimulation Mixture (BioLegend, Catalogue No. 423303) and a Protein Transporter Inhibitor (BD Biosciences, Catalogue No. 554724) for 4–6 h, after which they were collected for flow cytometry analysis to detect IFN‐γ, TNF‐α, and GzmB. To assess proliferation, MDSCs were cocultured with carboxyfluorescein succinimidyl ester (CFSE, Med Chem Express, Catalogue No. HY‐10627) labeled CD8^+^ T cells for 72 h. Finally, the cells were harvested and analyzed by flow cytometry to quantify CFSE^+^CD8^+^ T cells.

### 4.7 RNA‐Seq Profiling, qPCR

Total RNA was extracted using the Eastep Super Total RNA Extraction Kit (Promega, Catalogue No. LS1040). Subsequently, RNA reverse transcription was performed by preparing a 20 µL reverse transcription reaction system utilizing the PrimeScript RT kit (Takara, Catalogue No. RR037Q). Real‐time fluorescence quantitative PCR was conducted with the synergetic binding reagent (SYBR) qPCR Master Mix (Takara, Catalogue No. RR820Q), with the mouse Actin primer serving as the reference gene. The efficiencies of all qPCR assays adhered to the Minimum Information for Publication of Quantitative Real‐Time PCR Experiments (MIQE) guidelines. For RNA‐seq analysis, a total of 3 µg of RNA per sample was utilized as the input material for RNA sample preparation. The resulting products were purified and quantified using a Bioanalyzer 2100 system (Agilent, Palo Alto, CA, USA) with Agilent High Sensitivity DNA Analysis. Finally, the libraries were sequenced on the NovaSeq 6000 platform (Illumina Inc., San Diego, CA, USA).

### 4.8 Western Blot

Cytoplasmic protein lysates were prepared using a radioimmunoprecipitation assay (RIPA) lysis buffer that contained phenylmethylsulfonyl fluoride (PMSF; Beyotime, Catalogue No. ST506) and a phosphatase inhibitor (MCE, Catalogue No. HY‐K0021). Following protein quantification with the bicinchoninic acid (BCA) Protein Assay Kit (Beyotime, Catalogue No. P0012), the samples were separated by electrophoresis on 4%–12% Bis–Tris polyacrylamide gel electrophoresis (PAGE) and subsequently transferred to polyvinylidene fluoride (PVDF) membranes for further analysis. The membranes were blocked with 5% skimmed milk in triple buffered saline with Tween‐20 (TBST) for 2 h at room temperature, washed three times with TBST, and then incubated overnight at 4°C with a specific primary antibody solution at the appropriate concentration. Afterward, the membranes were incubated with a suitable IgG‐horseradish peroxidase (HRP)‐labeled secondary antibody (1:5000, Beyotime) in TBST for 1 h at room temperature. Antigens were visualized using chemiluminescence with Pierce Enhanced Chemiluminescence (ECL) West (Invitrogen, Catalogue No. A45917), and membrane‐bound immunocomplexes were detected using the ChemiDoc imaging system (Bio‐Rad). In our Western blot data, we calculated and normalized the ratios of target proteins to internal references in an Excel table, after analyzing the grayscale values of all protein bands using ImageJ software, based on consistent assay parameters. The first protein expression value from each group of bands was designated as a standard value of 1.00, with subsequent data representing the relative expression of the target proteins in the experimental group compared to the control group. The analyzed data are presented directly below the corresponding bands of the target molecules in the figure.

### 4.9 Flow Cytometry

Single‐cell suspensions of tissues were prepared through mechanical dispersion using the Meltenyi Biotec Gentle Magnetic‐activated Cell Sorting (MACS) Octo 8 and subsequent enzymatic digestion. For surface staining, cells were labeled with the appropriate antibodies (refer to the Supporting Information Table for various antibody staining protocols) for 30 min at 4°C in Ca^2+^/Mg^2+^‐free PBS supplemented with 2% FBS. In the case of single‐cell suspensions derived from tissues, an erythrocyte lysate procedure was conducted using erythrocyte lysate. For intracellular staining, surface‐labeled cells were fixed with Cytofix/Cytoperm solution (BD Biosciences, Catalogue No. 554714), washed with Perm/Wash Buffer 1 ×, and subsequently labeled with intracellular antibodies (see Supporting Information Table for different antibody staining protocols). The 2′,7′‐dichlorodihydrofluorescein diacetate (DCFH‐DA) kit (Beyotime, Catalogue No. S0035S) was utilized to detect reactive ROS. Samples of CH25H, ARG1, iNOS, PD‐L1, IFN‐γ, TNF‐α, GzmB, and ROS were analyzed alongside fluorescence minus one (FMO) controls from three different replicates, and mean fluorescence intensity (MFI) data were aggregated. All analyses were conducted using Flowjo V10 (Tree Star, Inc., Ashland, OR, USA) or Agilent software (Novocyte Advanteon).

### 4.10 Immunohistochemistry

The immunohistochemistry (IHC) protocols were performed in accordance with the previously established methodology [9]. An antibody targeting CH25H (1:300, Bioss, Catalogue No. bs‐41322R) was utilized for detection. The percentage of sample cells per field of view was quantified using ImageJ software (National Institutes of Health), and the AOD served as the evaluation metric for statistical analysis.

### 4.11 Cholesterol and 25HC Content Measurement by UPLC–MS/MS

MDSCs from *Ch25h* ^f/f^ and *Ch25h* ^f/f^Lyz2^Cre(±)^ mice were isolated and collected for the detection of cholesterol and its related metabolite, 25HC, utilizing UPLC–MS/MS, as previously described in references [9, 10]. The extracted samples were separated using an Acquity UPLC I‐Class system (Waters) and subsequently analyzed by mass spectrometry on an AB Sciex 6500 QTRAP. Data acquisition was performed using Analyst 1.6.2 software (Applied Biosystems).

### 4.12 Coimmunoprecipitation

The prepared cells were harvested for cellular proteins using Nonidet P 40 (NP40)/immunoprecipitation (IP) lysis buffer (Beyotime, Catalogue No. P0013F), which contained a protease inhibitor mixture with PMSF added at a ratio of 100:1. Whole cell lysates were then mixed with primary antibodies or normal mouse IgG and incubated overnight at 4°C on an overturned shaker. Following this incubation, preactivated magnetic beads were added to the mixture, which continued to be shaken overnight at 4°C. The samples were subsequently washed three to four times with PBS with Tween‐20 (PBST) wash buffer. Finally, the samples were eluted into an SDS sampling buffer and analyzed by protein blotting.

### 4.13 IF

For paraffin section samples: the dewaxed and antigenically repaired paraffin sections were placed in a microwave oven and treated with an ethylenediaminetetraacetic acid (EDTA) antigen repair solution. After natural cooling, the sections were transferred to PBS and subjected to decolorization on a shaker, being washed three times for 5 min each. The sections were then incubated in an osmotic buffer for 40 min. Following a 2‐h blocking period, the sections were incubated overnight at 4°C with the primary antibodies: CD33 (1:50, Santa Cruz, Catalogue No. sc‐19960) and CH25H (1:50, Bioss, Catalogue No. bs‐41322R). After three washes with PBS, the sections were incubated with a secondary antibody conjugated to a fluorescent dye (1:500). Finally, the sections were stained with 4′,6‐diamidino‐2′‐phenylindole (DAPI; Cell Signaling Technology, Catalogue No. 4083S) antifade reagent and visualized using a Leica fluorescence confocal microscope.

For frozen section samples: the tissues were washed in PBS for 30 min at room temperature and then fixed in 4% paraformaldehyde for 15 min. Following fixation, the tissues were washed several times with PBS, with each wash lasting 5 min. A circle was drawn around the tissue, after which a sealing solution was applied and allowed to set for 2 h at room temperature. After aspiration, CD33 primary antibody (1:500, 100 µL) was added and incubated at 37°C for 2 h, followed by three washes. The samples were then processed with a permeabilizer for 40 min and washed again. The CH25H or ARG1 (Cell Signaling Technology, Catalogue No. 93668S) primary antibody (1:200, 100 µL) was added, incubated overnight at 4°C, and washed three times after rewarming at room temperature for 30 min the following day. A secondary antibody (1:800, 200 µL) was added and incubated for 2 h at room temperature, followed by five washes. Finally, DAPI staining was performed, followed by five washes, coating with an antifluorescence quencher, and sealing. The samples were visualized using a Leica fluorescence confocal microscope.

### 4.14 ELISA

The ELISA kits used for the detection of IFN‐β (Shfksc, Catalogue No. F2124‐B) and CCL5 (Shfksc, Catalogue No. F30511‐B) in mouse MDSCs were obtained from Shanghai Fankew Biotechnology Company. The ELISA assays were conducted in accordance with the detailed instructions provided by the manufacturer.

### 4.15 Gene Set Enrichment Analysis (ssGSEA)

The CH25H and MDSC risk‐prognostic models were evaluated using four independent datasets from the GEO (https://www.ncbi.nlm.nih.gov/geo/) database: GSE39582, GSE17538, GSE87211, and GSE94104. Survival curves were generated with the survival R package and the survminer R package. To quantify the degree of infiltration of MDSCs in the samples, we employed the single sample ssGSEA algorithm to score MDSC‐related gene sets. ssGSEA is a nonparametric sorting method based on gene expression matrices, which calculates enrichment scores for specific gene sets independently for each sample. This method is particularly suitable for assessing the activity of specific functional states, such as immune cell infiltration. For our analyses, we utilized the “gsva()”function from the R language GSVA package (version 1.50.5), setting the method to “ssgsea” and inputting a preorganized set of MDSC marker genes as the reference gene set. This gene set can be derived from core marker genes that are highly expressed in MDSCs (e.g., human: CD33, CD14, CD15, S100 calcium‐binding protein A8 (S100A8), S100A9, etc.), as reported in authoritative literature, the ImmPort database, the Molecular Signatures Database (MSigDB) database, or experimental data. Ultimately, the algorithm generates a normalized enrichment score for each sample, referred to as the MDSC score. This score serves as a quantitative indicator of the characteristic activity of MDSCs in the sample, representing their relative abundance.

### 4.16 Statistical Analysis

The statistical analysis was performed using GraphPad Prism version 9.0 (GraphPad Software, San Diego, CA, USA). Experimental data are presented as mean ± standard error of the mean (SEM) values. A Shapiro–Wilk test was initially conducted to evaluate the normality of the data. Data were deemed normally distributed if the *p* value from the Shapiro–Wilk test exceeded 0.05. Following the normality assessment, a two‐tailed unpaired Student's *t*‐test was applied to compare the means of the two groups, given that the data were normally distributed. For studies involving multiple groups and tumor growth, a series of comparative analyses were conducted using one‐way analysis of variance (ANOVA), followed by the Bonferroni correction. The results of the specific statistical tests are indicated in the respective figures. A *p* value of less than 0.05 was regarded as statistically significant. The following symbols denote the levels of significance: ns indicates *p* > 0.05; * indicates *p* < 0.05; ** indicates *p* < 0.01; and *** indicates *p* < 0.001.

## Author Contributions

**Dongqin Zhou**: data organization, validation, visualization and analysis, writing original draft. **Yu Chen**: managing data, formal analysis, methodology. **Xudong Liu**: visualization. **Juan He**: data verification, investigation. **Luyao Shen**: methodology. **Yongpeng He**: resources. **Jiangang Zhang**: methodology. **Yu Zhou and Nan Zhang**: Survey. **Yanquan Xu**: methodology. **Juan Lei and Ran Ren**: Survey. **Huakan Zhao**: writing ‐ review and editing. **Xianghua Zeng**: project management, writing ‐ review, editing, funding access. **Yongsheng Li**: project administration, conceptualization, monitoring, writing ‐ review, editing, funding acquisition. All the authors reviewed the manuscript, shared feedback, and approved the manuscript in its final form.

## Ethics Statement

This collection adhered to the ethical guidelines outlined in the Declaration of Helsinki and received approval from the Institutional Review Board of Chongqing University Cancer Hospital (CZLS2022255‐A‐34). Written informed consent was obtained from all participants, including both patients and healthy individuals. All animal experimental protocols strictly complied with the National Institutes of Health guidelines for animal care and were approved by the Institutional Animal Care and Use Committee of Chongqing University Cancer Hospital, under the designated animal ethics number (CZLS2022255‐A‐34). Notably, both the animal experiments and the clinical study were approved under the same ethics approval number in the application year.

## Conflicts of Interest

The authors declare no conflicts of interest.

## Supporting information




**Supporting Figure 1**: The increased abundance of CH25H in tumor‐infiltrating MDSCs. (A) Biofiducial analysis reveals differences in CH25H levels between CRC tissues and normal tissues. (B) CH25H levels in bone marrow, spleen, and tumor tissue MDSCs in the subcutaneous B16‐F10 model, using bone marrow MDSCs as the control group (*n* = 3). (C) CH25H levels in bone marrow, spleen, normal liver tissue, and tumor tissue MDSCs in the Hepa1‐6 in situ hormonal model, using bone marrow MDSCs as the control group (*n* = 3). (D) CH25H levels in bone marrow, spleen, and tumor tissue MDSCs of the subcutaneous LLC model, using bone marrow MDSCs as the control group (*n* = 5). Data are expressed as mean ± SEM values. Statistical comparisons with controls indicate ns for no significant difference, *p < 0.05, **p < 0.01, and ***p < 0.001.
**Supporting Figure 2**: The immunosuppressive function of CH25H‐derived 25HC in regulating tumor‐associated MDSCs. (A) PCA of WT MDSCs treated with RPMI 1640 medium (control) or MC38‐TCM, alongside UPLC–MS/MS testing of the relative levels of free cholesterol‐related metabolites in these samples. (B, C) The protein levels of iNOS and PD‐L1 under varying concentrations of 25HC treatment, using DMSO as a control. (D) The levels of ROS in MDSCs following a 6h treatment with 25HC (0.5 µM) were assessed using the DCF‐DA probe, using DMSO as a control (*n* = 3). (E) The relative mRNA levels of *Nos2*, *Cd274*, and *Abca1* following treatment with different concentrations of 25HC, using DMSO as a control (*n* = 3). (F) MDSCs treated with 25HC (0.5 µM, 6 h), using DMSO as a control, in combination with MC38‐TCM were cocultured with activation‐stimulated CD8^+^ T cells (1:3 ratio) for 48 h to measure TNFα levels in T cells (*n* = 3). Data are expressed as mean ± SEM. Statistical comparisons indicate ns for no significant difference, *p < 0.05, **p < 0.01, ***p < 0.001.
**Supporting Figure 3**: Myeloid deletion of CH25H reduces MDSCs immunosuppression, an effect that can be reversed by the addition of 25HC. (A) Results of genotyping for *Ch25h* ^f/f^Lyz2^Cre^ mice. (B) UPLC–MS/MS analysis revealing changes in the relative content of free cholesterol and its metabolite, 25HC, in *Ch25h* ^f/f^Lyz2^Cre(±)^ MDSCs, with *Ch25h* ^f/f^ MDSCs serving as the control (*n* = 3). (C) Protein levels of CH25H were assessed using Western blot analysis following the induction of *Ch25h* ^f/f^Lyz2^Cre(±)^ MDSCs through in vitro treatment with RPMI1640 medium, with *Ch25h* ^f/f^ MDSCs serving as the control group. Data are presented as mean ± SEM values. Statistical comparisons indicate ns for no significant difference, *p < 0.05, **p < 0.01, and ***p < 0.001 when compared to controls.
**Supporting Figure 4**: The regulation of MDSCs by 25HC is contingent upon the cGAS–STING pathway. (A) qPCR was conducted to evaluate the gene expression levels of *Ccl5* and *Ifnb* in WT MDSCs following treatment with 25HC (0.5 µM). Additionally, the gene expression levels in *Ch25h* ^f/f^Lyz2^Cre(±)^ MDSCs were assessed, with *Ch25h* ^f/f^ MDSCs serving as the control (*n* = 3). (B) ELISA was performed to measure the levels of CCL5 and IFN‐β in WT MDSCs post‐treatment with 25HC (0.5 µM), as well as to observe the alterations in CCL5 and IFN‐β levels in *Ch25h* ^f/f^Lyz2^Cre(±)^ MDSCs, where *Ch25h* ^f/f^ MDSCs acted as the control (*n* = 5). Data are presented as mean ± SEM values. Statistical comparisons indicate ns for no significant difference, *p < 0.05, **p < 0.01, and ***p < 0.001 when compared to controls.
**Supporting Figure 5**: The deletion of CH25H inhibits ARG1 expression through the activation of the TBK1–RIPK3 complex. (A) An online analysis of the predicted protein interaction network involving TBK1 and RIPK3 can be accessed via the following link: https://cn.string‐db.org/cgi/network?taskId=b0JJ4L1Dr9I3&sessionId=b80EmG14CNJg. (B) A structural interactions plot of TBK1 and RIPK3 proteins is presented, with TBK1 depicted in pink and RIPK3 in yellow.
**Supporting Figure 6**: The role of CH25H in tumor progression through CRC‐infiltrating MDSCs in patients. (A) Immunofluorescence was employed to assess the infiltration rate of MDSCs and the expression levels of CH25H in MDSCs from patients at various stages of CRC. The fluorescence intensity was statistically analyzed using Image J (*n* = 4). (B) Immunofluorescence was utilized to evaluate the infiltration rate of MDSCs and the expression levels of ARG1 in MDSCs from patients with different stages of CRC, with fluorescence intensity statistically analyzed by Image J (*n* = 4). All of the above data use stage I as a control. Data are presented as mean ± SEM values. Statistical comparisons indicate ns for no significant difference, *p < 0.05, **p < 0.01, and ***p < 0.001 when compared to controls.
**Supporting Table 1**: Primers for RT‐qPCR.
**Supporting Table 2**: The key antibodies in this study.

## Data Availability

All data necessary to evaluate the conclusions presented in this paper are included within the main text and/or the Supporting Information. For RNA sequencing data, please access the website associated with the accession number GSE284266 at https://www.ncbi.nlm.nih.gov/bioproject. Furthermore, additional data supporting the results of this study can be requested from the corresponding author upon reasonable demand.
