# Supplementary material for: Cholesterol 25‐Hydroxylase Enhances Myeloid‐Derived Suppressor Cell (MDSC) Immunosuppression via the Stimulator of Interferon Genes (STING)‐Tank‐Binding Kinase 1 (TBK1)‐Receptor‐Interacting Protein Kinase 3 (RIPK3) Pathway in Colorectal Cancer
Source: MedComm (2020). 2025 Sep 27;6(10):e70411. doi: 10.1002/mco2.70411 (PMC12475974; doi:10.1002/mco2.70411)
Supplement: Supplementary file 1 — Supporting Figure 1: The increased abundance of CH25H in tumor‐infiltrating MDSCs. (A) Biofiducial analysis reveals differences in CH25H levels between CRC tissues and normal tissues. (B) CH25H levels in bone marrow, spleen, and tumor tissue MDSCs in the subcutaneous B16‐F10 model, using bone marrow MDSCs as the control group (n = 3). (C) CH25H levels in bone marrow, spleen, normal liver tissue, and tumor tissue MDSCs in the Hepa1‐6 in situ hormonal model, using bone marrow MDSCs as the control group (n = 3). (D) CH25H levels in bone marrow, spleen, and tumor tissue MDSCs of the subcutaneous LLC model, using bone marrow MDSCs as the control group (n = 5). Data are expressed as mean ± SEM values. Statistical comparisons with controls indicate ns for no significant difference, *p < 0.05, **p < 0.01, and ***p < 0.001. Supporting Figure 2: The immunosuppressive function of CH25H‐derived 25HC in regulating tumor‐associated MDSCs. (A) PCA of WT MDSCs treated with RPMI 1640 medium (control) or MC38‐TCM, alongside UPLC–MS/MS testing of the relative levels of free cholesterol‐related metabolites in these samples. (B, C) The protein levels of iNOS and PD‐L1 under varying concentrations of 25HC treatment, using DMSO as a control. (D) The levels of ROS in MDSCs following a 6h treatment with 25HC (0.5 µM) were assessed using the DCF‐DA probe, using DMSO as a control (n = 3). (E) The relative mRNA levels of Nos2, Cd274, and Abca1 following treatment with different concentrations of 25HC, using DMSO as a control (n = 3). (F) MDSCs treated with 25HC (0.5 µM, 6 h), using DMSO as a control, in combination with MC38‐TCM were cocultured with activation‐stimulated CD8+ T cells (1:3 ratio) for 48 h to measure TNFα levels in T cells (n = 3). Data are expressed as mean ± SEM. Statistical comparisons indicate ns for no significant difference, *p < 0.05, **p < 0.01, ***p < 0.001. Supporting Figure 3: Myeloid deletion of CH25H reduces MDSCs immunosuppression, an effect that can be reversed by [file MCO2-6-e70411-s001.pdf]

Cholesterol 25-hydroxylase enhances myeloid-derived suppressor cell (MDSC) immunosuppression *via* the stimulator of interferon genes (STING)-tank-binding kinase 1 (TBK1)-receptor-interacting protein kinase 3 (RIPK3) pathway in colorectal cancer

Dongqin Zhou[1#], Yu Chen[2#], Xudong Liu[3], Juan He[3], Luyao Shen[4], Yongpeng He[5], Jiangang Zhang[2], Yu Zhou[2], Nan Zhang[3], Yanquan Xu[6], Juan Lei[2], Ran Ren[3], Huakan Zhao[2]*, Xianghua Zeng[2]*, Yongsheng Li[1,2]*

1. The Second Affiliated Hospital and Yuying Children's Hospital of Wenzhou Medical University, Wenzhou, Zhejiang, 325000, China;
2. Department of Medical Oncology, Chongqing University Cancer Hospital, Chongqing 400030, China;
3. Chongqing University Cancer Hospital, School of Medicine, Chongqing University, Chongqing 400044, China
4. Department of Clinical Laboratory, Air Force Medical Center，Haidian District, Beijing;
5. Chongqing Key Laboratory of Translational Research for Cancer Metastasis and Individualized Treatment, Chongqing University Cancer Hospital & Chongqing Cancer Institute & Chongqing Cancer Hospital, Chongqing, 400030, China;
6. Clinical Medicine Research Center, Xinqiao Hospital, Army Medical University, Chongqing, 400037, China

***Corresponding author:** Yongsheng Li, E-mail: lys@cqu.edu.cn, Tel (Fax): 86-23-65079255; Xianghua Zeng, E-mail: zengxh0803@163.com; Huakan Zhao, E-mail: hk_zhao@cqu.edu.cn.

Supplementary Data for

Cholesterol 25-hydroxylase enhances myeloid-derived suppressor cell (MDSC) immunosuppression *via* the stimulator of interferon genes (STING)-tank-binding kinase 1 (TBK1)-receptor-interacting protein kinase 3 (RIPK3) pathway in colorectal cancer

Included:

Figures S1-S6

Table S1-S2

Supplementary Figures：

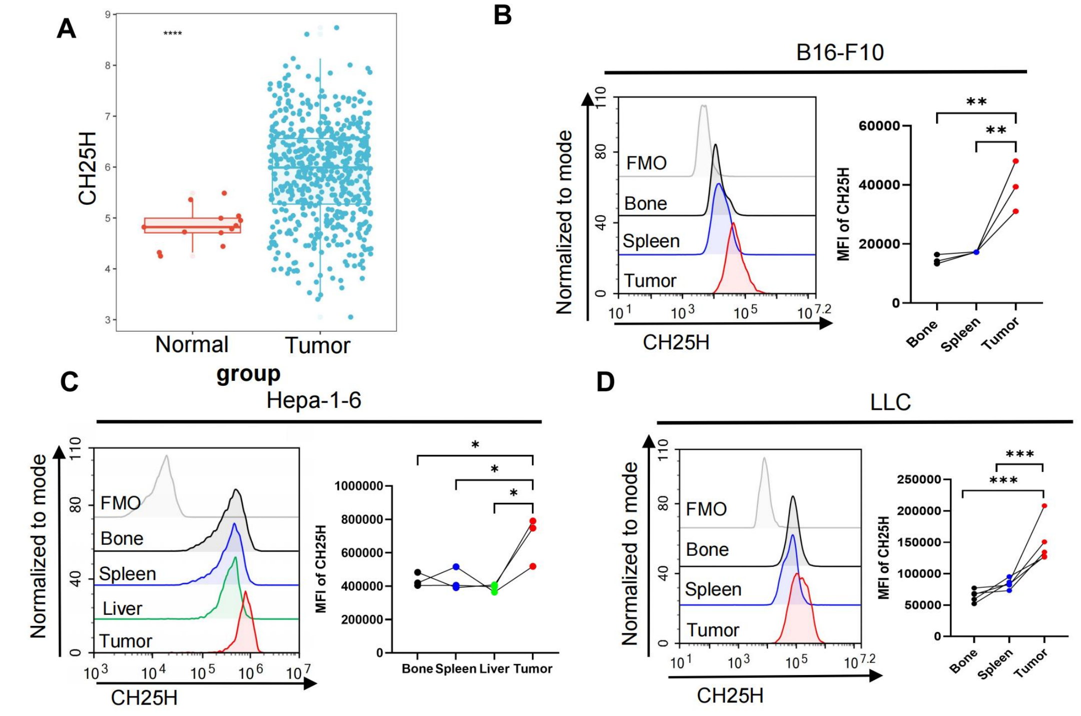

Figure S1. The increased abundance of CH25H in tumor-infiltrating MDSCs. (A) Biofiducial analysis reveals differences in CH25H levels between CRC tissues and normal tissues. (B) CH25H levels in bone marrow, spleen, and tumor tissue MDSCs in the subcutaneous B16-F10 model, using bone marrow MDSCs as the control group (n=3). (C) CH25H levels in bone marrow, spleen, normal liver tissue, and tumor tissue MDSCs in the Hepa1-6 *in situ* hormonal model, using bone marrow MDSCs as the control group (n=3). (D) CH25H levels in bone marrow, spleen, and tumor tissue MDSCs of the subcutaneous LLC model, using bone marrow MDSCs as the control group (n=5). Data are expressed as mean ± SEM values. Statistical comparisons with controls indicate ns for no difference, *p < 0.05, **p < 0.01, and ***p < 0.001.

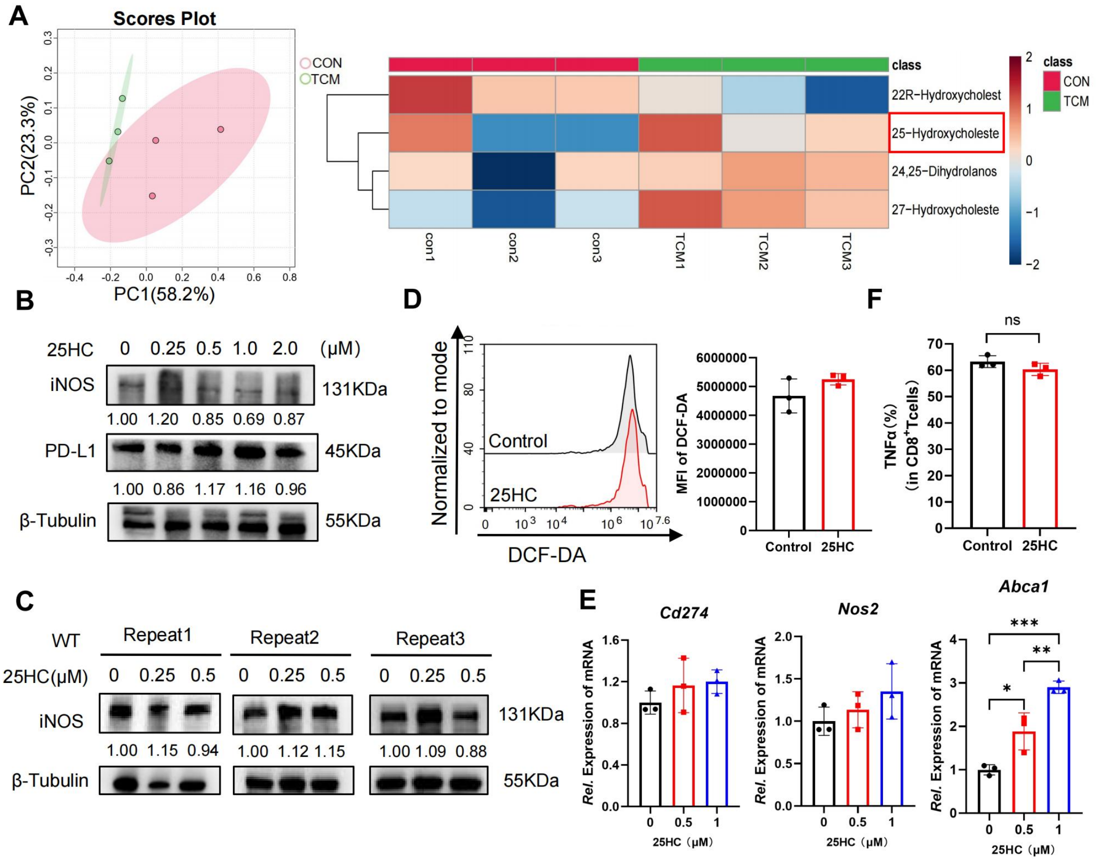

Figure S2. The immunosuppressive function of CH25H-derived 25HC in regulating tumor-associated MDSCs. (A) PCA of WT MDSCs treated with RPMI 1640 medium (control) or MC38 TCM, alongside UPLC-MS/MS testing of the relative levels of free cholesterol-related metabolites in these samples. (B) and (C) The protein levels of iNOS and PD-L1 under varying concentrations of 25HC treatment, using DMSO as a control. (D) The levels of ROS in MDSCs following a 6-hour treatment with 25HC (0.5 μM) were assessed using the DCF-DA probe, using DMSO as a control (n=3). (E) The relative mRNA levels of Nos2, Cd274, and Abca1 following treatment with different concentrations of 25HC, using DMSO as a control (n=3). (F) MDSCs treated with 25HC (0.5 μM, 6h), using DMSO as a control, in combination with MC38-TCM were co-cultured with activation-stimulated $CD8^+$ T cells (1:3 ratio) for 48 hours to measure TNFα levels in T cells (n=3). Data are expressed as mean ± SEM. Statistical comparisons indicate ns for no significant difference, *$p < 0.05$, **$p < 0.01$, ***$p < 0.001$.

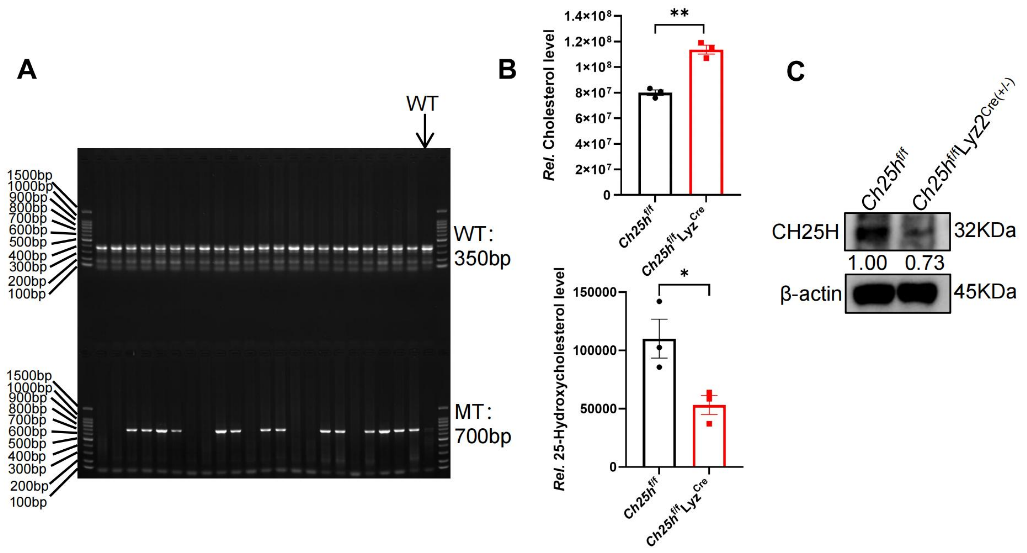

Figure S3. Myeloid deletion of CH25H reduces MDSC immunosuppression, an effect that can be reversed by the addition of 25HC. (A) Results of genotyping for *Ch25h*$^{f/f}$Lyz2^{Cre} mice. (B) UPLC-MS/MS analysis revealing changes in the relative content of free cholesterol and its metabolite, 25HC, in *Ch25h*$^{f/f}$Lyz2$^{Cre(+/-)}$ MDSCs, with *Ch25h*$^{f/f}$ MDSCs serving as the control (n=3). (C) Protein levels of CH25H were assessed using Western Blot analysis following the induction of *Ch25h*$^{f/f}$Lyz2$^{Cre(+/-)}$ MDSCs through *in vitro* treatment with RPMI-1640 medium, with *Ch25h*$^{f/f}$ MDSCs serving as the control group. Data are presented as mean ± SEM values. Statistical comparisons indicate ns for no difference, $*p < 0.05$, $**p < 0.01$, and $***p < 0.001$ when compared to controls.

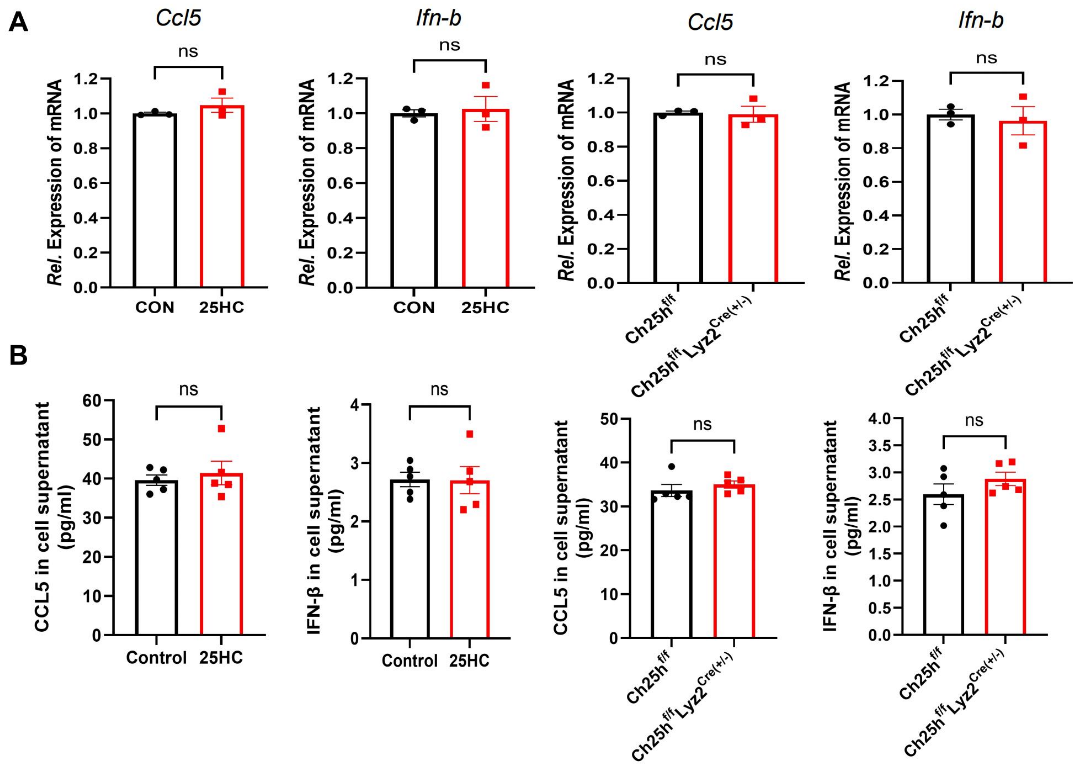

Figure S4. The regulation of MDSCs by 25HC is contingent upon the cGAS-STING pathway. (A) qPCR was conducted to evaluate the gene expression levels of *Ccl5* and *Ifnb* in WT MDSCs following treatment with 25HC (0.5 μM). Additionally, the gene expression levels in *Ch25h*$^{f/f}$Lyz2$^{Cre(+/-)}$ MDSCs were assessed, with *Ch25h*$^{f/f}$ MDSCs serving as the control (n=3). (B) ELISA was performed to measure the levels of CCL5 and IFN-β in WT MDSCs post-treatment with 25HC (0.5 μM), as well as to observe the alterations in CCL5 and IFN-β levels in *Ch25h*$^{f/f}$Lyz2$^{Cre(+/-)}$ MDSCs, where *Ch25h*$^{f/f}$ MDSCs acted as the control (n=5). Data are presented as mean ± SEM values. Statistical comparisons indicate ns for no difference, *$p < 0.05$, **$p < 0.01$, and ***$p < 0.001$ when compared to controls.

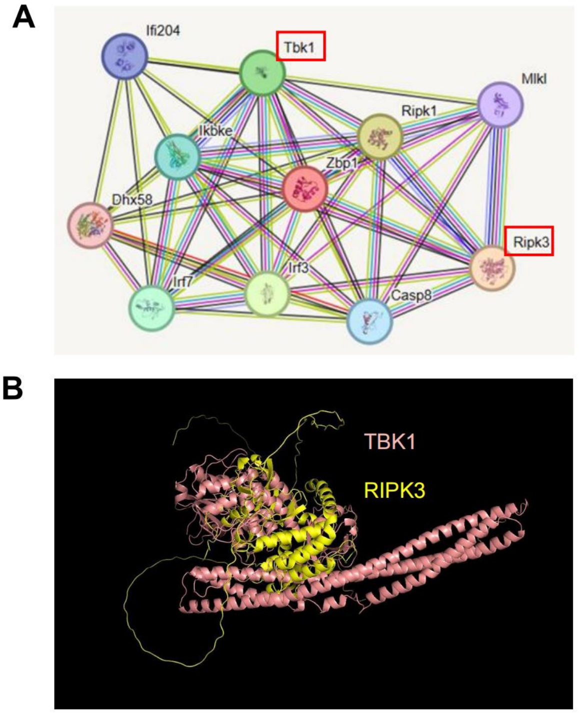

Figure S5. The deletion of CH25H inhibits ARG1 expression through the activation of the TBK1-RIPK3 complex. (A) An online analysis of the predicted protein interaction network involving TBK1 and RIPK3 can be accessed via the following link: https://cn.string-db.org/cgi/network?taskId=b0JJ4L1Dr9I3&sessionId=b80EmG14CNJg. (B) A structural interactions plot of TBK1 and RIPK3 proteins is presented, with TBK1 depicted in pink and RIPK3 in yellow.

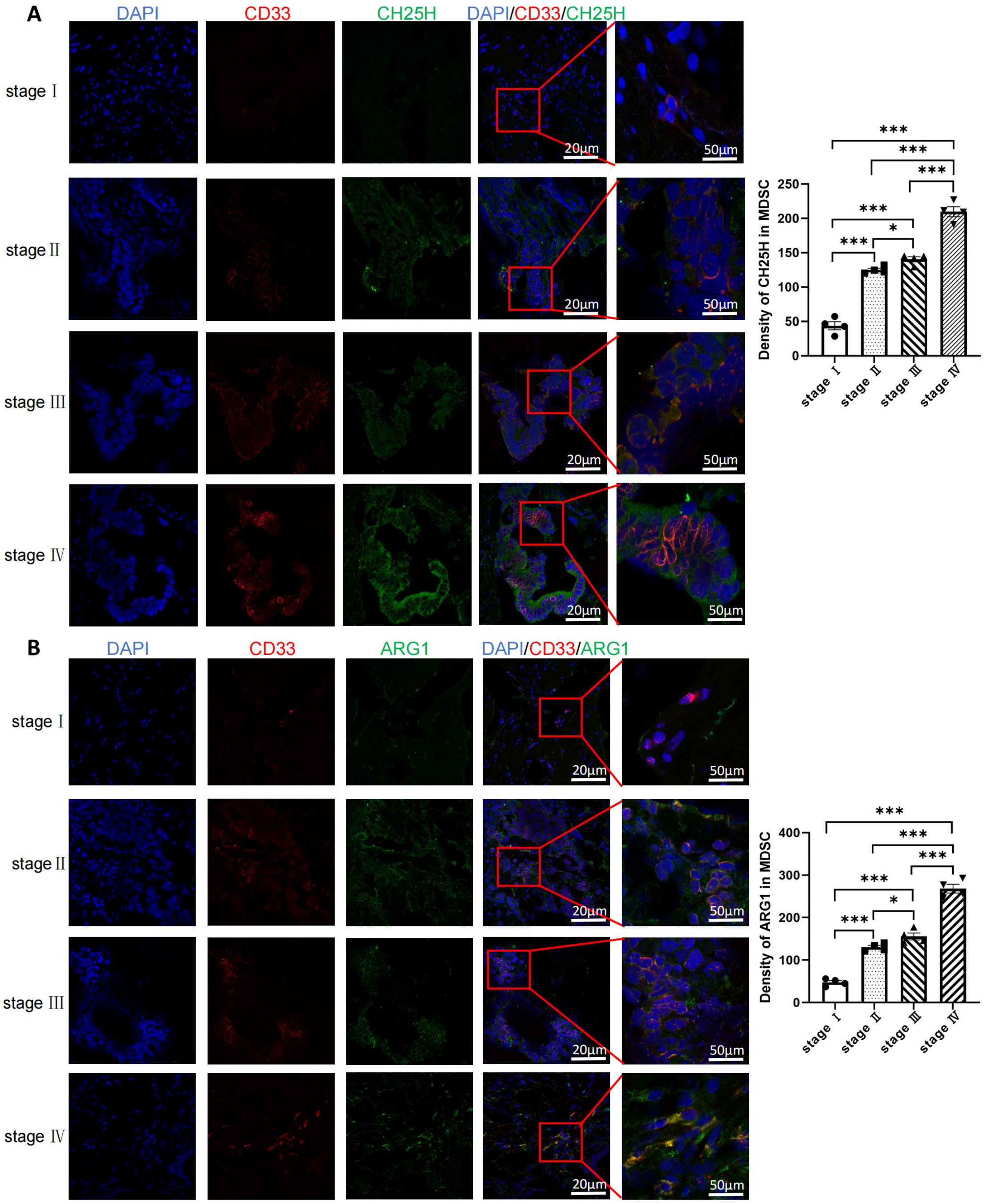

Figure S6. The role of CH25H in tumor progression through CRC-infiltrating MDSCs in patients. (A) Immunofluorescence was employed to assess the infiltration rate of MDSCs and the expression levels of CH25H in MDSCs from patients at various stages of CRC. The fluorescence intensity was statistically analyzed using Image J (n=4). (B) Immunofluorescence was utilized to evaluate the infiltration rate of MDSCs and the expression levels of ARG1 in MDSCs from patients with different stages of CRC, with fluorescence intensity statistically analyzed by Image J

(n=4). All of the above data use stage Ⅰ as a control. Data are presented as mean ± SEM values. Statistical comparisons indicate ns for no difference, *$p < 0.05$, **$p < 0.01$, and ***$p < 0.001$ when compared to controls.

Supplementary Tables:

Table S1. Primers for RT-qPCR.

	**Sequence of forward primer**	**Sequence of reverse primer**
**m-*β-actin***	TGACAGGATGCAGAAGGAGA	GTACTTGCGCTCAGGAGGAG
**m-*Arg1***	CATTGGCTTGCGAGACGTAGAC	GCTGAAGGTCTCTTCCATCACC
**m-*Ch25h***	CTACCGTTCGTGGTGCTGGA	CGGGAACACGAACACCAGGT
**m-*Lyz2Cre*-wt**	TTACAGTCGGCCAGGCTGAC	CTTGGGCTGCCAGAATTTCTC
**m-*Lyz2Cre*-mt**	CTTGGGCTGCCAGAATTTCTC	CCCAGAAATGCCAGATTACG
**m-*Sting***	GGTCACCGCTCCAAATATGTAG	CAGTAGTCCAAGTTCGTGCGA
**m-*Sting*-mt**	TCTCCCCATTCAGAAGCCACTTG	AGGCCAGCCCACTGTGATTGTAT
**m-*Sting*-wt**	CTGGGCTGGCAGAACACTCTAAG	CAGGCTGGCCACCAGAAAGAT
**m-*NOS2***	GAGACAGGGAAGTCTGAAGCAC	CCAGCAGTAGTTGCTCCTCTTC
**m-*Cd247***	GCTGAAGGTCTCTTCCATCACC	CCAAGGGTCCTTGTGAGTTCTG
**m-*Abca1***	GGAGCCTTTGTGGAACTCTTCC	CGCTCTCTTCAGCCACTTTGAG
**m-*Ccl5***	CCTGCTGCTTTGCCTACCTCTC	ACACACTTGGCGGTTCCTTCGA
**m-*Ifnb***	GCCTTTGCCATCCAAGAGATGC	ACACTGTCTGCTGGTGGAGTTC

Table S2. The key antibodies in this study.

ANTYBODIES	Supplier	IDENTIFIER
**β-actin**	Cell Signaling Technology	Cat. #8457S
**ARG1**	Cell Signaling Technology	Cat# 93668S; RRID: AB_2800207
**INOS**	Cell Signaling Technology	Cat#13120S
**PD-L1**	Proteintech	Cat#66248-1-Ig; RRID:AB_2756526
**ABCA1**	Origene	Cat# TA309555
**Mouse-Reactive STING Pathway Antibody Sampler Kit**	Cell Signaling Technology	Cat#16029T
**RIPK3**	Cell Signaling Technology	Cat# 95702S; RRID: AB_2721823
**p-RIPK3**	Cell Signaling Technology	Cat# 91702S
**CH25H**	Santa Cruz	sc-293256
**CH25H**	Bioss	bs-23620R
**CD33**	Santa Cruz	sc-19660
**Pacific Blue™anti-mouse/human CD11b**	Biolegend	Cat# 101224; RRID: AB_755986
**APC anti-mouse CD45**	Biolegend	Cat# 103112; RRID: AB_312977
**PE anti-mouse CD45**	Biolegend	Cat# 157604; RRID: AB_2876536
**FITC anti-mouse CD45**	Biolegend	Cat# 157214; RRID: AB_2894427
**PerCP anti-mouse Ly-6G/Ly-6C (Gr-1)**	Biolegend	Cat# 108426; RRID: AB_893557
**PE anti-human HLA-DR**	Biolegend	Cat# 307606; RRID: AB_314684
**Pacific Blue™ anti-mouse CD8a**	Biolegend	Cat# 100725; RRID: AB_493425
**APC anti-mouse CD8a**	Biolegend	Cat# 100712; RRID: AB_312751
**FITC anti-mouse Ly-6G/Ly-6C (Gr-1)**	Biolegend	Cat#108406; Clone no.RB6-8C5
**Purified Rat Anti-Mouse CD23**	BD Biosciences	Cat# 553136
**PerCP/Cyanine5.5 anti-mouse CD3ε Recombinant**	Biolegend	Cat#155704; Clone no.QA17A05
**FITC anti-mouse IFN-γ**	Biolegend	Cat#505806; Clone no.XMG1.2
**FITC anti-mouse TNF-α**	Biolegend	Cat#506304; Clone no.MP6-XT22
**Pacific Blue™ anti-human/mouse Granzyme B**	Biolegend	Cat#372218; Clone no.QA16A02
**FITC anti-human/mouse Granzyme B**	Biolegend	Cat# 372206; RRID: AB_2687030
